# TMEM63B channel is the osmosensor required for thirst drive of interoceptive neurons

**DOI:** 10.1038/s41421-023-00628-x

**Published:** 2024-01-03

**Authors:** Guolin Yang, Min Jia, Guizhou Li, Yan-Yu Zang, Yang-Yang Chen, Yue-Ying Wang, Shi-Yu Zhan, Shi-Xiao Peng, Guoqiang Wan, Wei Li, Jian-Jun Yang, Yun Stone Shi

**Affiliations:** 1 State Key Laboratory of Pharmaceutical Biotechnology, Department of Neurosurgery, Drum Tower Hospital Affiliated to Medical School of Nanjing University, Nanjing, Jiangsu China; 2 Department of Anesthesiology, Pain and Perioperative Medicine, First Affiliated Hospital of Zhengzhou University, Zhengzhou, Henan China; 3 Ministry of Education Key Laboratory of Model Animal for Disease Study, Model Animal Research Center, Nanjing University, Nanjing, Jiangsu China; 4 Guangdong Institute of Intelligence Science and Technology, Hengqin, Zhuhai, Guangdong China; 5 Present Address: Guangdong Institute of Intelligence Science and Technology, Hengqin, Zhuhai, Guangdong China

**Keywords:** Ion channel signalling, Nutrient signalling

## Abstract

Thirst plays a vital role in the regulation of body fluid homeostasis and if deregulated can be life-threatening. Interoceptive neurons in the subfornical organ (SFO) are intrinsically osmosensitive and their activation by hyperosmolarity is necessary and sufficient for generating thirst. However, the primary molecules sensing systemic osmolarity in these neurons remain elusive. Here we show that the mechanosensitive TMEM63B cation channel is the osmosensor required for the interoceptive neurons to drive thirst. TMEM63B channel is highly expressed in the excitatory SFO thirst neurons. TMEM63B deletion in these neurons impaired hyperosmolarity-induced drinking behavior, while re-expressing TMEM63B in SFO restored water appetite in TMEM63B-deficient mice. Remarkably, hyperosmolarity activates TMEM63B channels, leading to depolarization and increased firing rate of the interoceptive neurons, which drives drinking behavior. Furthermore, TMEM63B deletion did not affect sensitivities of the SFO neurons to angiotensin II or hypoosmolarity, suggesting that TMEM63B plays a specialized role in detecting hyperosmolarity in SFO neurons. Thus, our results reveal a critical osmosensor molecule for the generation of thirst perception.

## Introduction

Human bodies contain a large amount of water that accounts for ~60% of the body weight, which is constantly lost through evaporation, respiration and excretion, resulting in the risk of dehydration^1–4^. To achieve fluid homeostasis, thirst motivates animals to adjust adaptive water-seeking behavior and hormonal responses to resist the elevation in systemic osmolarity^1,2,5–8^.

The lamina terminalis (LT) is the principal brain structure responsible for monitoring homeostatic signals of internal fluid balance and responsible for initiation of thirst perception, which is referred as thirst center^1,2,5,6,9^. There are three nuclei in the LT, the subfornical organ (SFO), the organic vasculosum lamina terminalis (OVLT) and the median preoptic nucleus (MnPO), all of which are anatomically interconnected. Among them, MnPO cannot directly sense blood osmolarity and is considered to be an integration center; the SFO and OVLT lie outside the blood-brain barrier, thus are able to directly sense the circulatory osmotic information^1,2,6,9^. The interoceptive neurons in SFO and OVLT are intrinsically osmosensitive, and function as osmoreceptors; their firing rates increase in response to the elevation of systemic osmolarity^10–14^. Activation of these excitatory neurons motivates drinking behavior whereas inhibition suppresses drinking, thus they are referred as thirst neurons^1,2,9,15^. The transduction of the osmotic information has been postulated to be mediated by stretch-activated ion channels located on the plasma membranes of SFO and OVLT interoceptive neurons through monitoring the osmolarity-induced cell-volume changes^1,2,16^. Previous studies have suggested that TRPV1 channel serves as a potential osmosensor in OVLT^13,14^. However, the deletion of TRPV1 in mice did not affect thirst behavior or hypertonicity-induced FOS activation in the thirst center^17–19^, indicating the involvement of other, as yet unidentified molecules in this crucial role. The identity of the osmosensor in thirst neurons thus remains to be one of the key unsolved issues toward understanding the thirst perception^1,2^.

In principle, this osmosensory protein, whether it is an ion channel, receptor or other molecule, is expected to fulfill certain criteria. First, it should be expressed in the interoceptive neurons in the SFO and/or OVLT. Second, it should directly sense the internal osmolarity and couple to neuronal activity. Last, deleting it from the thirst center would impair thirst responses in animals while re-expressing it should rescue thirst behavior.

TMEM63 family of cation channels, the homologs of plant OSCAs in animals^20^, are recently characterized to be osmo-^21–24^ and mechano-sensitive^25–27^. The physiological functions of TMEM63s are beginning to be revealed. In Drosophila, TMEM63 channel detects environmental humidity and food texture preference^28,29^. In mammals, TMEM63A mutations cause infant-onset transient hypomyelination^30^. TMEM63C regulates the glomerular filtration barrier function^31^. Our recent work found that TMEM63B is an osmosensor required for survival of cochlear outer hair cells upon hearing onset^21^. In this study, we found that TMEM63B deletion in SFO neurons impaired drinking behavior in mice. TMEM63B in SFO neurons was activated by hyperosmotic stress, which leads to depolarization and increased firing rate of these neurons. The neuronal sensitivity to hyperosmolality was eliminated by TMEM63B deletion. However, TMEM63B deletion did not affect the neuronal sensitivity to angiotensin-II or hypoosmolality. Therefore, TMEM63B channel in the SFO interoceptive neurons is the osmosensor detecting systemic hyperosmolality, playing a central role in the generation of thirst perception.

## Result

### TMEM63B is abundantly expressed in SFO

Our first hypothesis was that the unidentified osmosensor would be expressed in the excitatory interoceptive neurons in SFO. Recently, single-cell RNA sequencing (scRNA-seq) has been performed in the LT thirst center^32^. Thus, we analyzed the gene expression levels of putative osmosensitive or mechanosensitive ion channels or receptors in major cell classes in the SFO using the scRNA-seq data (Supplementary Fig. S1). One of the candidate genes, *Tmem63b*, was most enriched in the SFO excitatory neurons (Supplementary Fig. S1).

**Fig. 1:**
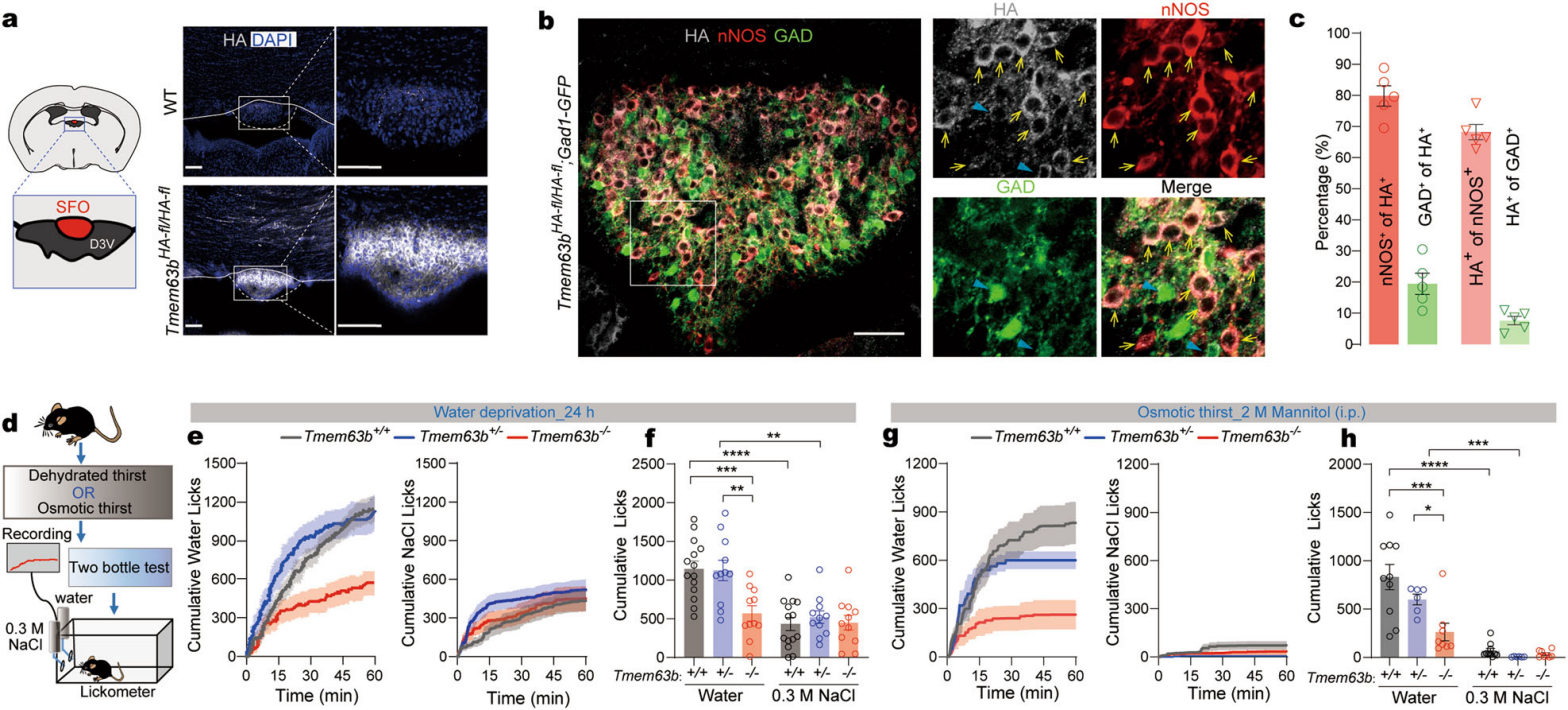
TMEM63B is expressed in SFO and TMEM63B deletion impairs water appetite. **a** Left: schematic illustration showing SFO position in brain. D3V, dorsal 3rd ventricle. Right: immunochemical detection of HA expression in the SFO of WT and *Tmem63b*^*HA-fl/HA-fl*^ mice. Scale bars, 100 μm. **b** Immunochemical detection of HA, nNOS and GAD in the SFO of *Tmem63b*^*HA-fl/HA-fl*^*;Gad1-GFP* mice. Scale bar, 50 μm. Arrowheads and arrows showing that cells containing HA signal overlapped with GAD (indicated by blue arrowheads) and nNOS (indicated by yellow arrow). **c** Percentages of HA-positive cells with nNOS or GAD, or percentages of nNOS- or GAD-positive cells with HA (*n* = 3 mice, 5 slices). **d** Experimental design to induce dehydrated thirst (water deprivation for 24 h) or osmotic thirst (intraperitoneal injection of 2 M Mannitol). **e** Time course of water licking (left) or 0.3 M NaCl licking (right) in the two-bottle test under water deprivation. **f** Quantification of water and NaCl licking for 60 min after water deprivation in *Tmem63b*^*+/+*^, *Tmem63b*^*+/−*^ and *Tmem63b*^*−/−*^ mice (*n* = 11−14 mice in each experiments). **g** Time course of licking after i.p. injection of 2 M mannitol. **h** Quantification of licking after mannitol injection in *Tmem63b*^*+/+*^ (*n* = 10 mice), *Tmem63b*^*+/−*^ (*n* = 6 mice) and *Tmem63b*^*−/−*^ mice (*n* = 8 mice). Data are means ± SEM, *P < 0.05, **P < 0.01, ***P < 0.001, ****P < 0.0001; two-way repeated-measures ANOVA with Holm-Šídák post-hoc analysis for **f** and **h**.

We then validated TMEM63B expression in the SFO using *Tmem63b*^*HA-fl/HA-fl*^ mice, in which hemagglutinin (HA) was tagged at the N-terminal of TMEM63B and two LoxP segments were flanked for the induction of conditional knockout of *Tmem63b* gene^21^. Immunofluorescence showed robust HA signal in the SFO (Fig. 1a). ~80% of HA-positive cells overlapped with nNOS, marker of the excitatory neurons in the SFO (Fig. 1b, c). The remaining minor population of HA-positive neurons appeared to be inhibitory neurons as shown in the SFO of *Tmem63b*^*HA-fl/HA-fl*^*;Gad1-GFP* mice (Fig. 1b, c), and the HA signal was relatively weaker than that in excitatory neurons (arrows in Fig. 1b), consistent with the scRNA-seq data.

### TMEM63B is required for thirst

Recently, the TMEM63B channel was demonstrated to be mechano-^25–27^ and osmo-sensitive^21,22,24^. As it is highly expressed in SFO neurons, we speculated that TMEM63B might serve as the osmosensor for thirst. To test this hypothesis, we first examined dehydration-induced drinking behavior in *Tmem63b*^*−/−*^ mice^21^. After 24 h of water deprivation, mice were placed individually in lickometer cages containing two bottles filled with 0.3 M NaCl (salt) and pure water, respectively (Fig. 1d). Water intake by *Tmem63b*^*−/−*^ mice was approximately half of that by wild-type (WT, *Tmem63b*^*+/+*^) or *Tmem63b*^*+/−*^ animals while salt intake was comparable among them (Fig. 1e, f). Water deprivation causes both osmotic thirst and hypovolemic thirst that would trigger water and salt appetite through differential pathways^1,2,5,32^. To verify that the impaired water appetite was related to osmotic thirst, we intraperitoneally (i.p.) injected 2 M mannitol (10 µL/g) hypertonic solution to induce an osmotic challenge^1,32^. After injection of mannitol, the salt intake by WT mice was negligible (Fig. 1g, h), indicating that osmotic thirst drove water appetite rather than salt appetite. Again, *Tmem63b*^*−/−*^ mice displayed reduced water appetite (Fig. 1g, h), indicating impaired thirst response to internal hyperosmolarity. Furthermore, to assess whether the effect of TMEM63B on water consumption is caused by the central mechanism, we analyzed the drinking behavior of the *Tmem63b*^*HA-fl/HA-fl*^*;Nestin-Cre* mouse line^22,33,34^, in which TMEM63B was conditionally knocked out (cKO) in the nervous system (Supplementary Fig. S2a). Water intake was similarly reduced in the TMEM63B cKO mice upon water deprivation or induction of osmotic thirst (Supplementary Fig. S2b–e), suggesting that TMEM63B regulates thirst through a central mechanism.

### TMEM63B in SFO is essential for water appetite

**Fig. 2:**
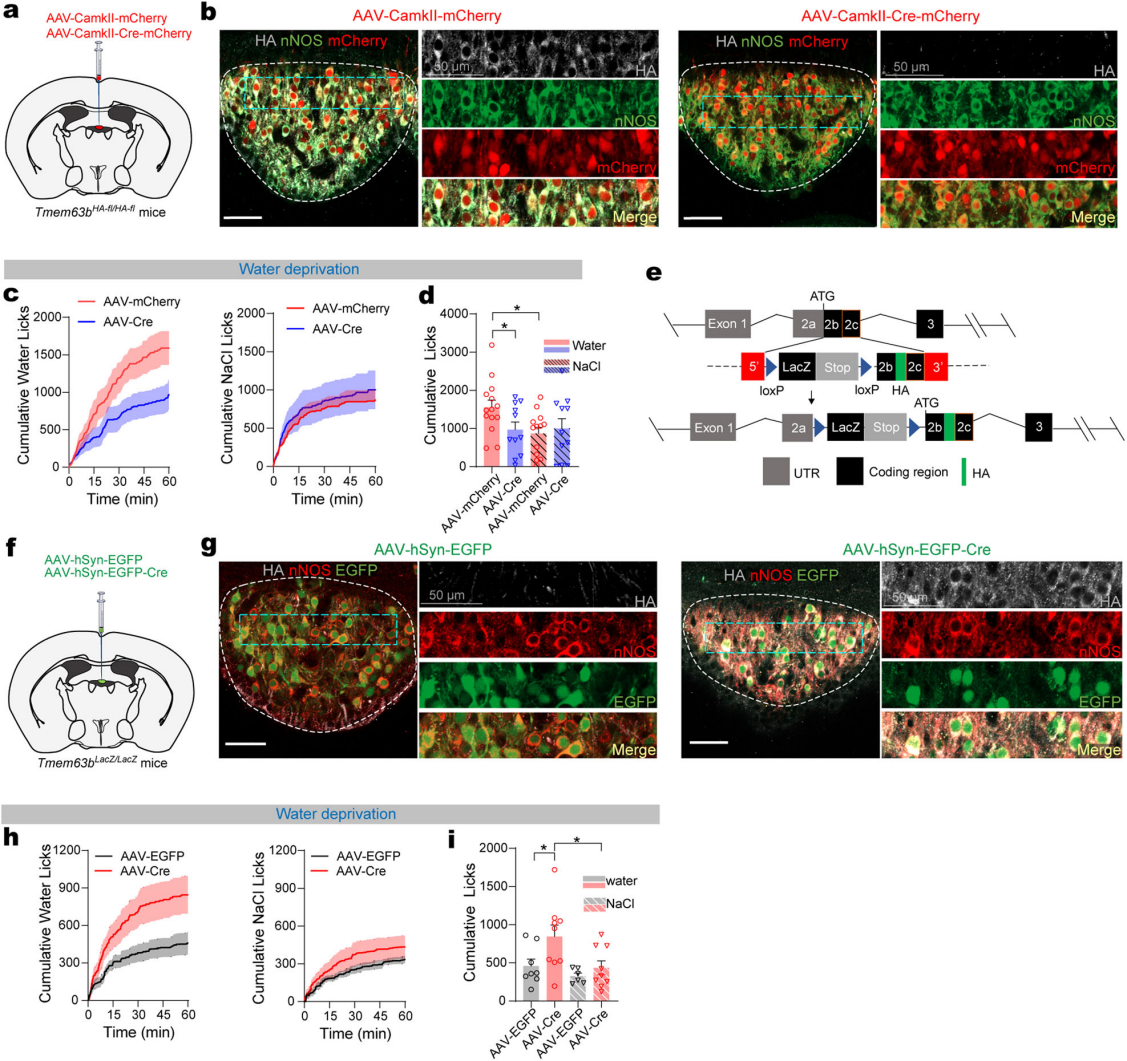
TMEM63B in SFO is essential for thirst appetite. **a** Schematic of virus injection into SFO of *Tmem63b*^*HA-fl/HA-fl*^ mice to produce SFO-specific TMEM63B deletion. **b** Representative images validating knockout of TMEM63B by immunostaining HA. Scale bars, 50 μm. **c** Time course of water licking and 0.3 M NaCl licking under water deprivation in *Tmem63b*^*HA-fl/HA-fl*^ mice injected with AAV-Cre or AAV-mCherry virus. **d** Quantification of licking in AAV-Cre- (*n* = 14 mice) and AAV-mCherry- (*n* = 12 mice) injected animals. **e** Schematic illustrating the strategy used to generate *Tmem63b*^*LacZ*^ mouse line. **f** Schematic of virus injection into SFO of *Tmem63b*^*LacZ/LacZ*^ mice to produce SFO-specific TMEM63B rescue. **g** Representative images validating re-expression of TMEM63B by immunostaining HA. Scale bars, 50 μm. **h** Time course of water licking or 0.3 M NaCl licking under water deprivation in *Tmem63b*^*LacZ/LacZ*^ mice injected with AAV-Cre or AAV-EGFP. **i** Quantification of licking in AAV-Cre- (*n* = 8 mice) and AAV-EGFP- (*n* = 9 mice) injected animals. Data are means ± SEM, *P < 0.05, **P < 0.01; two-way repeated-measures ANOVA with Holm-Šídák post-hoc analysis for **d** and **i**.

To reveal the physiological role of TMEM63B in SFO, we examined the effect of SFO-specific deletion of TMEM63B on drinking behavior through injecting an adeno-associated virus (AAV) carrying Cre-recombinase directly into the SFO of *Tmem63b*^*HA-fl/HA-fl*^ mice (Fig. 2a). Successful deletion of TMEM63B in the SFO was validated by immunostaining of HA-TMEM63B (Fig. 2b). Two-bottle experiments showed that AAV-Cre-injected mice had reduced water intake but not salt consumption compared to AAV-mCherry-injected controls after water deprivation (Fig. 2c, d; Supplementary Fig. S3), illustrating that TMEM63B in SFO thirst neurons is required for water appetite.

We next wondered whether re-expressing TMEM63B in SFO could rescue the water appetite of the TMEM63B-deficient mice. For this purpose, we generated a conditional rescue mouse line, *Tmem63b*^*LacZ*^, by inserting a *loxP-LacZ-polyA-loxP* fragment in front of the coding region of *Tmem63b* gene (Fig. 2e). An HA-tag was also inserted after the signal peptide sequence of TMEM63B to facilitate detection of TMEM63B protein. Mice carrying this allele express LacZ driven by the *Tmem63b* promoter which efficiently terminated TMEM63B expression (Supplementary Fig. S4a). We then rescued TMEM63B expression in the SFO by injecting AAV-hSyn-EGFP-Cre directly into the SFO of *Tmem63b*^*LacZ/LacZ*^ mice (Fig. 2f). Immunofluorescence results demonstrated successful removal of β-galactosidase and re-expression of HA-TMEM63B in the SFO (Fig. 2g; Supplementary Fig. S4b). Consequently, TMEM63B expression was only restored in the SFO but not in other tissues (Supplementary Fig. S5). After dehydration, AAV-hSyn-Cre-injected mice had an increased water intake compared to AAV-hSyn-EGFP-injected control mice (Fig. 2h, i), reaching to ~74% of WT mice (Fig. 1e). Together, these results demonstrate that TMEM63B in the SFO neurons is both necessary and sufficient for generation of thirst perception.

### SFO^TMEM63B^ activity drives thirst behavior

We next generated a *Tmem63b*^*Cre*^ mouse line to directly examine the physiological role of TMEM63B-expressing SFO neurons (SFO^TMEM63B^), in which iCre-PolyA was inserted into the start codon ATG of *Tmem63b* gene, thereby the expression of iCre is driven by the endogenous *Tmem63b* promoter (Supplementary Fig. S6a). Meanwhile, the insertion of iCre-PolyA fragment disrupted the expression of *Tmem63b* gene, therefore the *Tmem63b*^*Cre/Cre*^ mice were essentially TMEM63B knockout (KO) while *Tmem63b*^*Cre/+*^ mice were similar to the *Tmem63b*^*+/−*^ mice (Supplementary Fig. S6b). We then investigated the effects of SFO^TMEM63B^ activities on thirst using optogenetic and chemogenetic approaches in freely behaving animals. Optogenetic or chemogenetic activation of the SFO^TMEM63B^ neurons immediately triggered thirst and prompted voracious water drinking under water satiated condition (Supplementary Fig. S6c−k), while chemogenetic silencing of the SFO^TMEM63B^ neurons abolished dehydration- or osmotic stress-induced water drinking, but not salt intaking (Supplementary Fig. S6l−o). Importantly, activation of SFO^TMEM63B^ neurons in dehydrated mice still led to overdrinking of water, but not the salt solution (Supplementary Fig. S6j). These results demonstrated that activation of the TMEM63B-expressing SFO neurons specifically drives water intake.

**Fig. 3:**
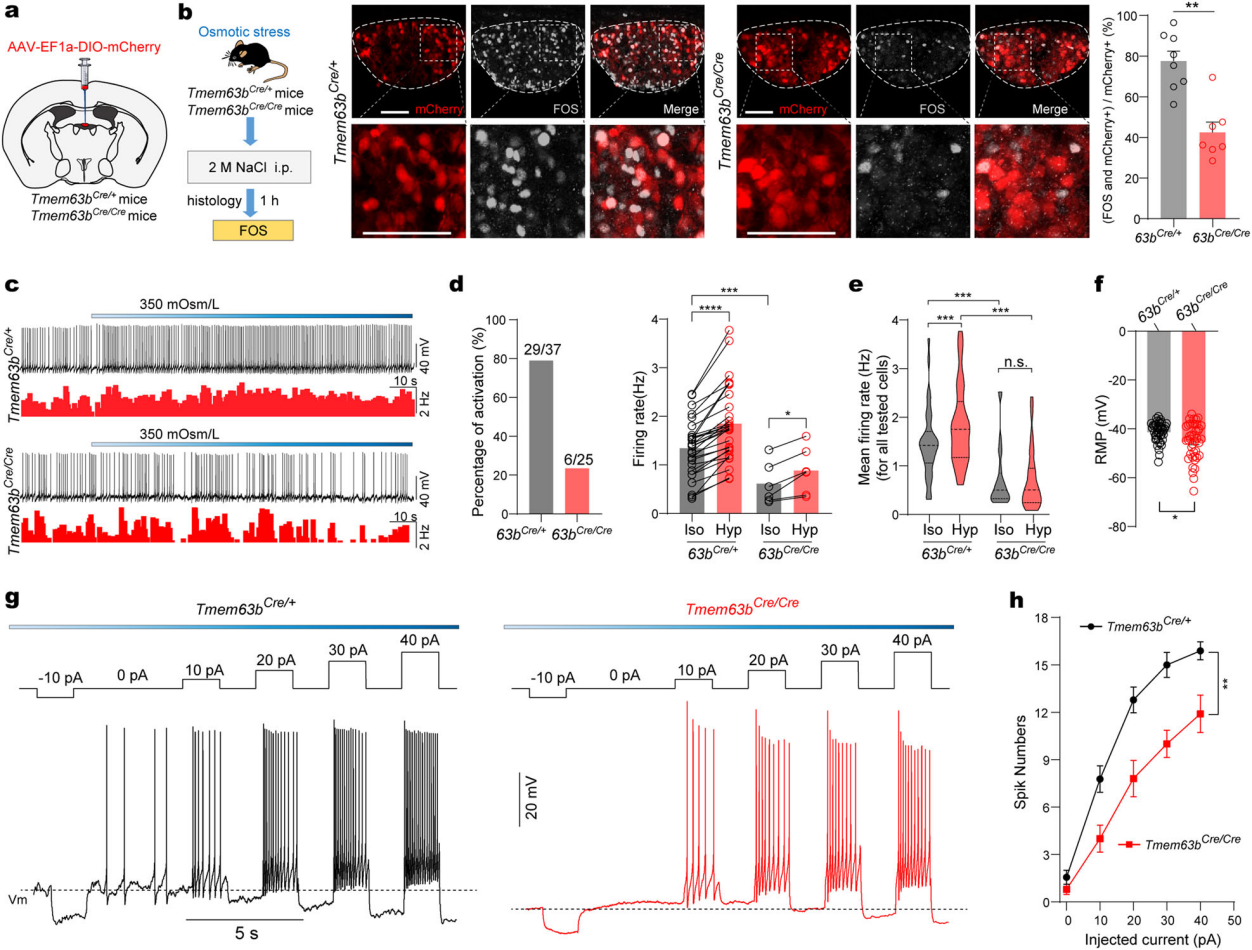
TMEM63B is required for osmotic activation of SFO^TMEM63B^ neurons. **a** Schematic of viral labeling of SFO^TMEM63B^ neurons. **b** Left: experimental design to excite SFO^TMEM63B^ neurons. Right: FOS immunoreactivity showing NaCl (i.p.)-activated SFO^TMEM63B^ neurons. Scale bars, 50 μm. Quantification: *n* = 8 mice for *Tmem63b*^*Cre/+*^ mice and *n* = 7 for *Tmem63b*^*Cre/Cre*^ mice. **c** Representative traces of the effect of hypertonic solution on the AP firing of SFO^TMEM63B^ neurons. Lower traces show firing rates. **d** Left: percentages of activated neurons after applying hypertonic solution (for *Tmem63b*^*Cre/+*^: *n* = 37 neurons from 16 mice, for *Tmem63b*^*Cre/Cre*^: *n* = 25 neurons from 9 mice). Right: firing rates of activated SFO^TMEM63B^ neurons from *Tmem63b*^*Cre/+*^ mice (*n* = 29 neurons, left) and *Tmem63b*^*Cre/Cre*^ mice (*n* = 6 neurons, right) shown in left, before (Iso) and after hypertonic stimulation (Hyp). **e** Mean firing rate for all tested neurons. **f** RMP in SFO^TMEM63B^ neurons from *Tmem63b*^*Cre/+*^ or *Tmem63b*^*Cre/Cre*^ mice. **g** Representative spike pattern of SFO^TMEM63B^ neurons in response to depolarizing currents. Current steps in the trace were made in 10-pA increments from –10 pA to +40 pA. V_m_, the membrane potential before applying injecting current. **h** The relationship between spike and injected current by plot spike-current curves for each SFO^TMEM63B^ neuron from *Tmem63b*^*Cre/+*^ neurons (*n* = 9) and *Tmem63b*^*Cre/Cre*^ neurons (*n* = 10). Data are means ± SEM, **P < 0.01, ***P < 0.001, ****P < 0.0001, n.s., no significant; two-tailed unpaired *t*-test for **b**, **f**, **h**; two-tailed paired *t*-test for **d**; two-way repeated-measures ANOVA with Holm-Šídák post-hoc analysis for **e**.

We next examined the role of TMEM63B in hyperosmolarity-induced neuronal activity of SFO^TMEM63B^ neurons. An AAV-DIO-mCherry virus was injected into the SFO of *Tmem63b*^*Cre/+*^ and *Tmem63b*^*Cre/Cre*^ mice to label SFO^TMEM63B^ neurons (Fig. 3a). Injection of hypertonic NaCl induced FOS expression in mCherry-labeled neurons, which was reduced in *Tmem63b*^*Cre/Cre*^ mice by about 50% compared to *Tmem63b*^*Cre/+*^ mice (Fig. 3b). We then examined SFO^TMEM63B^ response in brain slices; using this preparation we can block synaptic inputs; thus, indirect effects can be excluded. For hypertonic stimulus, we used solution with osmolarity at 350 mOsm/L, which is equivalent to the elevated blood osmolality caused by either water deprivation or salt challenge^32,35^. In brain slices, TMEM63B knockout reduced the cytosolic Ca^2+^ increase of SFO^TMEM63B^ neurons in response to the hypertonic solution, in the presence of blockers of AMPA, NMDA, and GABA receptors (synaptic blockers) (Supplementary Fig. S7a–f).

Whole-cell recording of mCherry-labeled SFO^TMEM63B^ neurons in acute brain slices showed spontaneous firing activity (Fig. 3c–e). When the perfusion solution was switched from an isotonic solution (300 mOsm/L) to a hypertonic solution (350 mOsm/L adjusted by mannitol) in the presence of synaptic blockers, 78% (29/37 cells tested) of SFO^TMEM63B^ neurons from *Tmem63b*^*Cre/+*^ mice had increased firing rates (Fig. 3d). In contrast, only 24% (6 of 25) cells showed similar response in *Tmem63b*^*Cre/Cre*^ mice (Fig. 3d). When all neurons were analyzed, the mean firing rate of *Tmem63b*^*Cre/Cre*^ neurons was not significantly altered (Fig. 3e). In addition, the spontaneous firing rates of SFO^TMEM63B^ neurons from *Tmem63b*^*Cre/Cre*^ mice were lower than that of *Tmem63b*^*Cre/+*^ mice under isotonic condition (Fig. 3e). The resting membrane potential (RMP) of these neurons from *Tmem63b*^*Cre/Cre*^ mice was more hyperpolarized (Fig. 3f). The hyperpolarized RMP suggested reduced neuronal excitability. Indeed, injection of depolarizing currents induced less firing in TMEM63B-deleted neurons compared to control neurons (Fig. 3g, h). Collectively, these results demonstrated that TMEM63B deletion reduced the excitability of SFO neurons and impaired their activation by a hypertonic stimulus.

### TMEM63B mediates intrinsic hyperosmolarity-sensing of the SFO^TMEM63B^ neurons

**Fig. 4:**
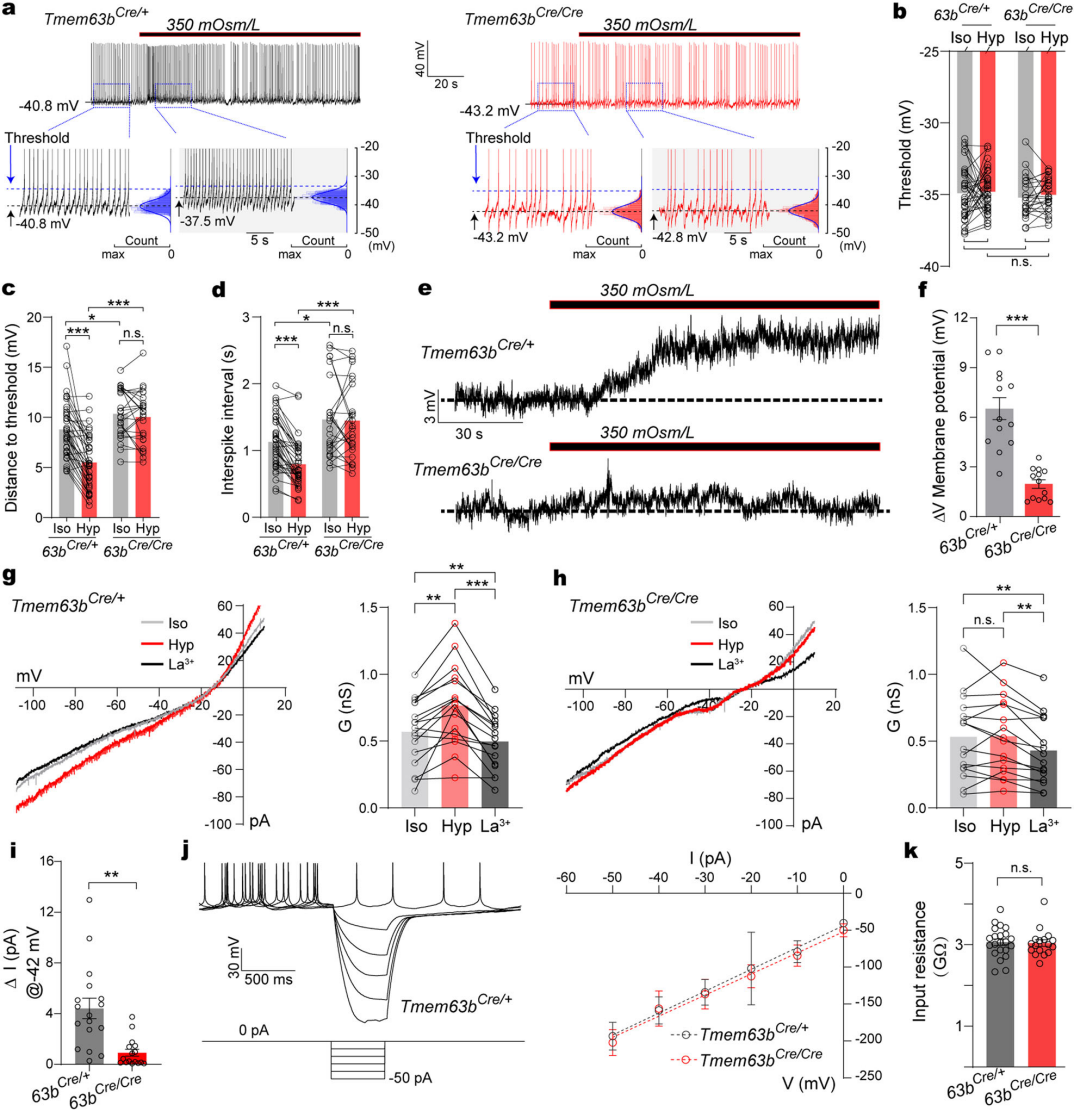
TMEM63B contributes to the osmosensory transduction of SFO^TMEM63B^ neurons in response to hyperosmolarity. **a** Top: schema of the action potential (AP) firing pattern of SFO^TMEM63B^ neurons from *Tmem63b*^*Cre/+*^ (left) mice and *Tmem63b*^*Cre/Cre*^ mice (right) before and after hypertonic stimulation. Bottom: AP are clipped for clarity. Black dashed lines show membrane potential; membrane potential was obtained from voltage histograms (blue gaussian curve). Threshold (blue dashed lines), AP threshold, was determined at the point where the voltage trace reached 10 mV/ms. **b** Quantification of the threshold from *Tmem63b*^*Cre/+*^ neurons (*n* = 37) and *Tmem63b*^*Cre/Cre*^ neurons (*n* = 25) before and after hypertonic stimulation. **c** Subthreshold depolarization (distance to threshold) before (Iso) and after hypertonic stimulation (Hyp) from *Tmem63b*^*Cre/+*^ mice (*n* = 37) and *Tmem63b*^*Cre/Cre*^ mice (*n* = 25). **d** The changes of interspike interval of SFO^TMEM63B^ neurons from *Tmem63b*^*Cre/+*^ mice (*n* = 37) and *Tmem63b*^*Cre/Cre*^ mice (*n* = 25) before and after hypertonic stimulation. **e** Representative voltage depolarization of SFO^TMEM63B^ neurons in response to hypertonic solution in the presence of TTX and synaptic blockers. **f** Quantification of the depolarized membrane potential from *Tmem63b*^*Cre/+*^ neurons (*n* = 13) and *Tmem63b*^*Cre/Cre*^ neurons (*n* = 14) after hypertonic stimulation (Hyp). **g** Left: representative currents recorded with ramp voltage protocol from a *Tmem63b*^*Cre/+*^ neuron. Iso, 300 mOsm/L; Hyp, 350 mOsm/L; La^3+^, 0.1 mM LaCl_3_ with hyperosmolarity. Right: quantification of slope conductance from –60 mV to –80 mV (*n* = 17). **h** Same as **g** but from *Tmem63b*^*Cre/Cre*^ mice (*n* = 16). **i** Increased current at RMP (–42 mV) in response to hyperosmolarity, calculated from **g**, **h**. **j** Left: representative voltage deflections from a *Tmem63b*^*Cre/+*^ neuron in response to hyperpolarizing currents (top), current steps (bottom) in the trace were made in 10-pA increments from –50 pA to 0 pA. Right: averaged current-voltage (I–V) plot across all tested neurons. **k** Input resistance calculated from the slop of *I*–*V* plot shown in **j** (*n* = 22 neurons for *Tmem63b*^*Cre/+*^, 17 for *Tmem63b*^*Cre/Cre*^). Data are means ± SEM, *P < 0.05, **P < 0.01, ***P < 0.001, n.s., no significant; two-tailed unpaired *t*-test for **f**, **i**, **k**; two-tailed paired *t*-test for **g**, **h** and two-way repea*t*ed-measures ANOVA with Holm-Šídák post-hoc analysis for **b**–**d**.

We analyzed subthreshold dynamics of the tonic firing of SFO neurons to understand how TMEM63B deletion blunted their response to hypertonic stress. These SFO^TMEM63B^ neurons showed tonic firing, with membrane potential spontaneously depolarized to reach action potential threshold (Fig. 4a). The action potential threshold was not changed by TMEM63B deletion or hypertonic stress (Fig. 4a, b). Since the RMP was hyperpolarized upon TMEM63B deletion, the amplitudes of subthreshold depolarization were larger in *Tmem63b*^*Cre/Cre*^ than in *Tmem63b*^*Cre/+*^ neurons (Fig. 4a, c). Perfusion of hypertonic solution significantly depolarized the membrane potential which further reduced the amplitudes of subthreshold depolarization in *Tmem63b*^*Cre/+*^ (Fig. 4a, c). These effects on membrane potential and subthreshold depolarization were absent in *Tmem63b*^*Cre/Cre*^ neurons (Fig. 4a, c). As a result, the interspike interval was shorter in *Tmem63b*^*Cre/+*^ than *Tmem63b*^*Cre/Cre*^ neurons (Fig. 4a, d). Hyperosmolarity further reduced the interspike interval in *Tmem63b*^*Cre/+*^ but not *Tmem63b*^*Cre/Cre*^ neurons (Fig. 4d). These subthreshold analyses indicated that TMEM63B channels not only contribute to membrane potential at basal condition but also mediate the hyperosmolarity-induced depolarization in SFO^TMEM63B^ neurons, which is the key function of a sensor molecule. To further verify this observation, we measured the RMP in the presence of TTX to block action potential (Fig. 4e). Again, hyperosmotic stress depolarized membrane potential of *Tmem63b*^*Cre/+*^ neurons by about 6 mV, which was abolished by TMEM63B deletion (Fig. 4f).

We further asked whether hyperosmolarity-induced depolarization is directly caused by TMEM63B channel activity. To address this question, we recorded whole-cell currents in SFO^TMEM63B^ neurons with hypertonic stress (Fig. 4g, h). Increasing osmolarity significantly increased the slope conductance of *Tmem63b*^*Cre/+*^ neurons, which was reversed by La^3+^, a non-selective cation channel blocker (Fig. 4g); hyperosmotic stimulation was unable to change the mean slope conductance of *Tmem63b*^*Cre/Cre*^ neurons (Fig. 4h). Notably, in *Tmem63b*^*Cre/+*^ neurons, hyperosmotic stress induced around 4 pA inward currents at –42 mV, the RMP of these SFO neurons (Fig. 4g, i). We wondered whether this seemingly small current had a capability of depolarizing the membrane potential to evoke firing. To address this issue, we measured input resistance of SFO^TMEM63B^ neurons which was ~3 GΩ (Fig. 4j, k), similar to the value of 2.5 GΩ reported in rat SFO neurons^36^. Such a big input resistance would compensate for the small currents to generate a relatively large depolarization. According to Ohm’s law, 4 pA is theoretically large enough to generate the experimentally recorded depolarization. Therefore, these results illustrated that TMEM63B activity mediates the osmosensory transduction of SFO thirst neurons.

### TMEM63B does not affect AngII-sensitivity of the SFO neurons

**Fig. 5:**
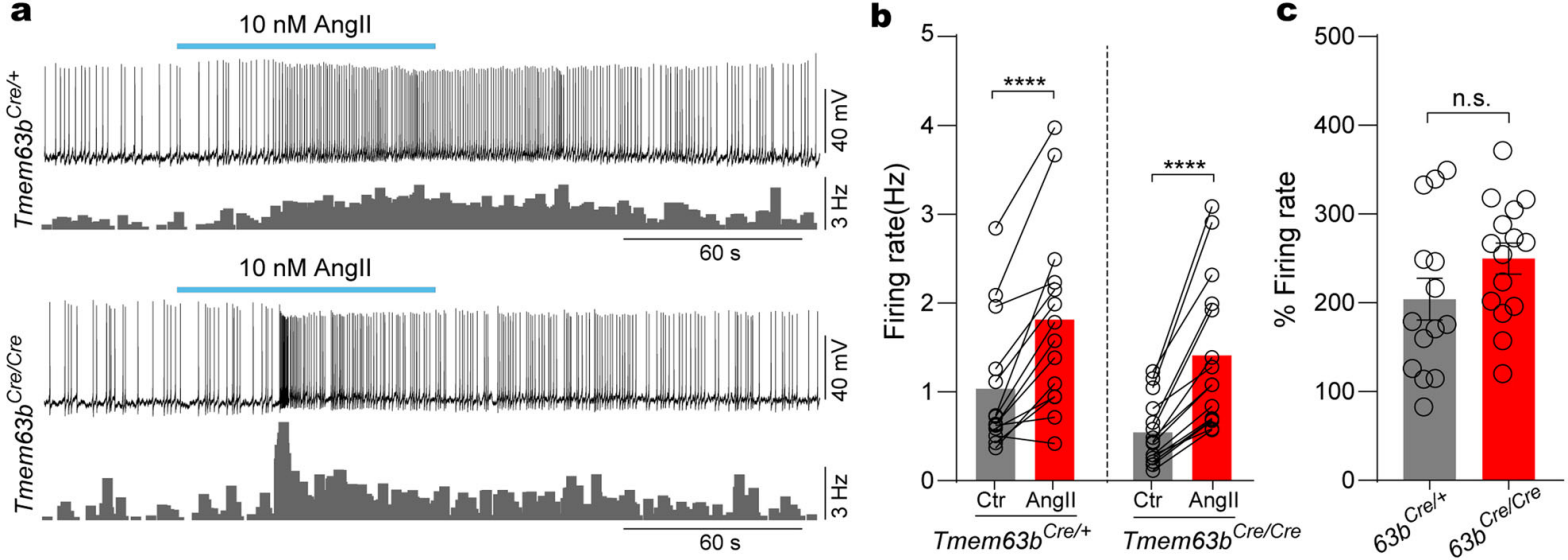
TMEM63B deletion does not affect AngII activation of SFO^TMEM63B^ neurons. **a** Representative traces of the effect of AngII on the AP firing of SFO^TMEM63B^ neurons. Lower traces show firing rates. **b** Firing rates for SFO^TMEM63B^ neurons from *Tmem63b*^*Cre/+*^ mice (*n* = 14 neurons) and *Tmem63b*^*Cre/Cre*^ mice (*n* = 15 neurons), before (Ctr) and during AngII stimulation. **c** The activation ratio of firing rate after AngII stimulation shown in **b**. Data are means ± SEM, ****P < 0.0001; n.s., no significant; two-tailed paired *t*-test for **b** and two-tailed unpaired *t*-test for **c**.

Electrophysiological recording demonstrated that the excitability of SFO thirst neurons is reduced by TMEM63B deletion. Could this be the common reason that TMEM63B KO neurons failed to respond to stimuli including hypertonic stress? To test this possibility, we examined the response of SFO neurons to the peptide hormone angiotensin II (AngII). With intensive expression of angiotensin II type 1 receptor (Agtr1a receptor)^15,32,37^ (Supplementary Fig. S1), SFO neurons are activated by plasma AngII^1,2,38^. Application of AngII (10 nM) evoked identical GCaMP signal in SFO^TMEM63B^ neurons in acute slices prepared from *Tmem63b*^*Cre/+*^ and *Tmem63b*^*Cre/Cre*^ mice (Supplementary Fig. S8a–f). Furthermore, the AngII-induced firing activity in *Tmem63b*^*Cre/Cre*^ neurons was comparable to that in *Tmem63b*^*Cre/+*^ neurons (Fig. 5a–c), demonstrating that TMEM63B deletion did not affect the responses of SFO thirst neurons to the AngII signal. Thus, these data demonstrated that TMEM63B deletion specifically impaired the sensitivity of SFO^TMEM63B^ neurons to hypertonic stress, but not hormones.

### TMEM63B does not affect SFO^TMEM63B^ sensitivity to hypoosmolality

**Fig. 6:**
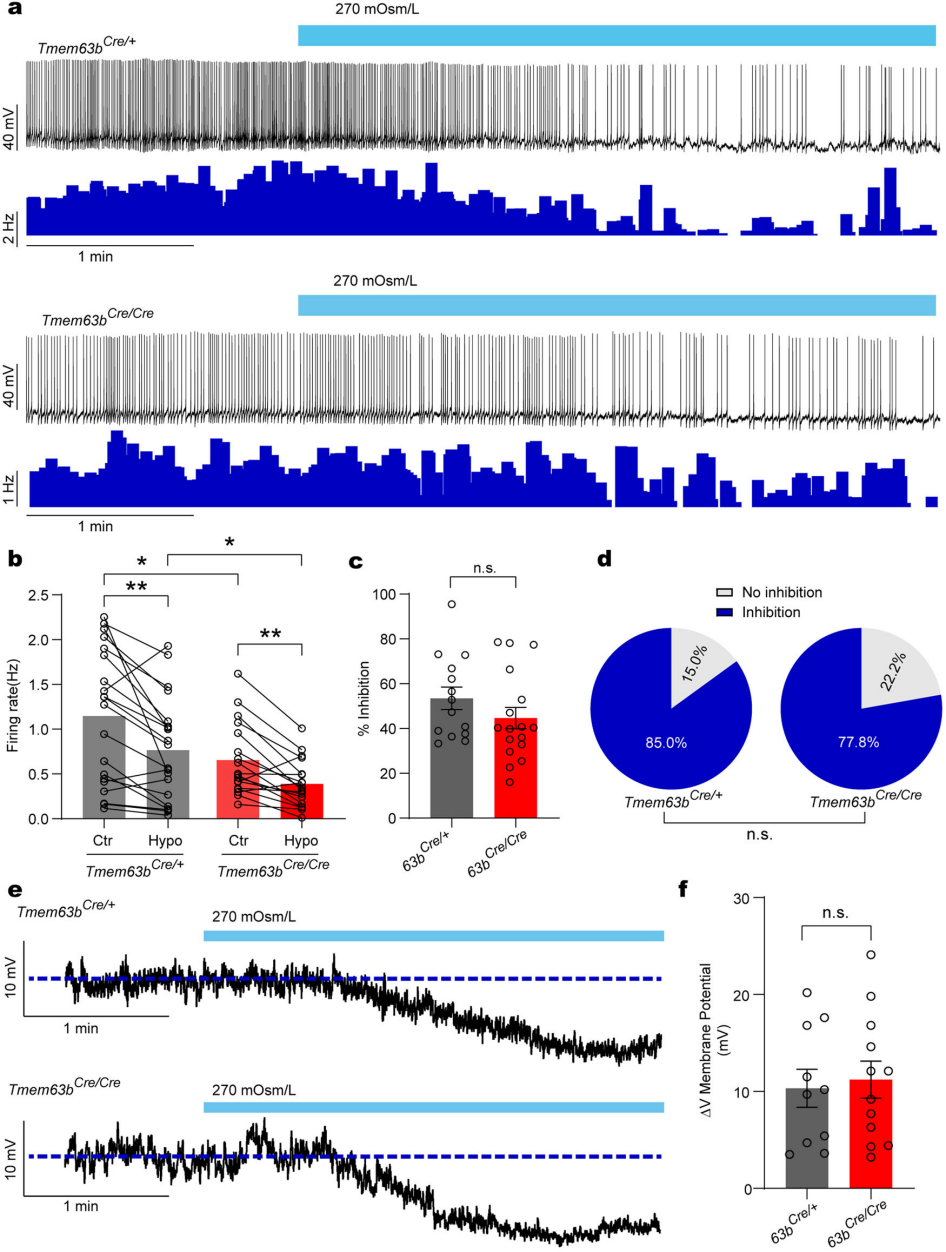
Hypoosmolarity reduces SFO^TMEM63B^ neuronal activity. **a** Representative traces of the effect of hypotonic solution on the AP firing of SFO^TMEM63B^ neurons. Lower traces show firing rates. **b** Quantification of firing rates of SFO^TMEM63B^ neuron from *Tmem63b*^*Cre/+*^ mice (*n* = 20 neurons, left) and *Tmem63b*^*Cre/Cre*^ mice (*n* = 18 neurons, right), before (Iso) and after hypotonic stimulation (Hypo). **c** The inhibition ratio of firing rate after hypotonic stimulation shown in **b**. Cells without inhibition are excluded. **d** Percentages of neurons displaying different responses after hypotonic stimulation shown in **b**. **e** Representative voltage fluctuation of SFO^TMEM63B^ neurons in response to hypotonic solution in the presence of TTX and synaptic blockers. **f** Quantification of the hyperpolarized membrane potential from *Tmem63b*^*Cre/+*^ neurons (*n* = 10) and *Tmem63b*^*Cre/Cre*^ neurons (*n* = 12) after hypotonic stimulation. Data are means ± SEM, *P < 0.05, **P < 0.01, n.s., no significant; two-tailed unpaired *t*-test for **c**, **f**; two-tailed paired *t*-test for **b**; χ^2^ test for **d**.

We have recently reported that recombinant TMEM63B is more sensitive to hypoosmotic stress than to hyperosmotic stimulus^21^. We wondered whether TMEM63B in SFO excitatory neurons can mediate a response to hypoosmotic signal. Whole-cell recording was used to examine the response of SFO^TMEM63B^ neurons to hypoosmotic solutions (270 mOsm/L) in acute brain slices (Fig. 6a). Hypoosmolarity reduced firing rate in majority (17/20 cells tested; ~85%) of *Tmem63b*^*Cre/+*^ neurons (Fig. 6b–d) accompanied by a large hyperpolarization in membrane potential (Fig. 6e, f), suggesting that SFO^TMEM63B^ neurons could directly detect hypoosmotic signal. Strikingly, the response to hypoosmotic stress was not different in *Tmem63b*^*Cre/cre*^ neurons (Fig. 6a–f). These observations suggested that either TMEM63B channel in SFO neurons is insensitive to hypotonic stimulus or hypotonicity-induced TMEM63B channel activity is insufficient to change membrane potential and firing rates.

## Discussion

In this study, we provided compelling evidence that TMEM63B is an osmosensor in SFO for generation of thirst perception. Firstly, TMEM63B is highly expressed in SFO excitatory neurons. The HA-TMEM63B signal in the SFO of *Tmem63b*^*HA-fl/HA-fl*^ mice mostly overlaps with nNOS-marked excitatory neurons, the putative thirst neurons^2,35,37^. The expression pattern of *Tmem63b* in the SFO is consistent with scRNA-seq expression data^32^. Secondly, using a number of TMEM63B deletion models including *Tmem63b*^−/−^, TMEM63B cKO in the nervous system or virus-based specific deletion of TMEM63B in SFO, we consistently observed that the water appetite is impaired. Thirdly, TMEM63B channels in the SFO neurons are activated by hypertonic stimulations, leading to membrane depolarization which couples to the firing rates of SFO excitatory neurons or SFO^TMEM63B^ neurons. Lastly, SFO-specific re-expressing TMEM63B restores drinking behavior in TMEM63B-deficient mice. These data demonstrate that TMEM63B in the SFO serves as the thirst-driving molecule.

Osmosensory transduction, the conversion of osmotic stimuli into electrical signals, is a fundamental process in the initiation of thirst perception. Using electrophysiological recordings, we showed that the SFO interoceptive neurons have tonic firing activity, with membrane potential spontaneously depolarized to reach action potential threshold. The RMP is about –42 mV. The input resistance is ~3 GΩ. The relatively high RMP and large input resistance indicate that the K^+^ channel activity is low in these neurons. We found that hypertonic stimulus induces La^3+^-sensitive inward currents in the SFO interoceptive neurons that are largely eliminated with TMEM63B deletion, demonstrating that the TMEM63B channels in SFO neurons are activated by hyperosmolarity. Although the current appears to be small, it can generate significant depolarization and increase the firing rate as these neurons have a large input resistance. Interestingly, TMEM63B deletion also causes ~3 mV hyperpolarization of the RMP, which leads to reduced excitability of these neurons. This observation suggests that TMEM63B in SFO neurons might have basal activity. Without TMEM63B-specific antagonists, we are unable to measure this putative TMEM63B tonic current. According to Ohm’s law, only 1–2 pA is required to generate 3 mV membrane potential shift due to the large input resistance. Taken together, we found evidence that TMEM63B not only contributes to the sensitivity to hyperosmolality (depolarization induced by hyperosmolarity) but also regulates the basal firing activity (changes in RMP) in SFO neurons. Deletion of TMEM63B does not affect the excitation by the peptide hormone AngII in these neurons, indicating that the reduced basal activity does not generally affect the neuronal sensitivity to stimuli. Impaired hyperosmolality-induced increases in firing rates of the TMEM63B-deficient SFO thirst neurons should attribute to their impaired sensitivity to hyperosmolarity.

We also characterized the sensitivity of SFO^TMEM63B^ neurons to hypoosmotic stimulus. We found that hypoosmotic stress hyperpolarizes 85% of SFO^TMEM63B^ neurons, and causes reduction of firing rate. These effects, however, are not changed by TMEM63B deletion. We speculate that the hypotonicity-induced hyperpolarization might be attributed to the activities of some K^+^ channels. We have recently reported that recombinant TMEM63B is more sensitive to hypoosmotic stress than to hyperosmotic stimulus^21^. It should be noted that experimental conditions are dramatically different between recombinant system (600 mOsm for hyperosmolarity and 170 mOsm for hypoosmolarity)^21^ and neurons (350 mOsm and 270 mOsm, respectively). Our data suggest that TMEM63B in SFO neurons is dramatically more sensitive to hyperosmotic stress than the recombinant TMEM63B. While the exact mechanism is unclear, differences in the expression levels or cellular environments, such as neuron-specific auxiliary subunits or binding partners, may contribute to the distinct sensitivity of TMEM63B in SFO neurons and cell lines. It is worth noting that differences in phenotypes of ion channels between recombinant systems and in vivo settings are not uncommon. For example, co-assembly with KCNE1 switches TMEM16A, a calcium activated chloride channel, from a calcium-dependent to a voltage-dependent ion channel^39^.

TMEM63 family of cation channels are animal homologs of the plant OSCA channels^20^. Multiple studies have demonstrated that the OSCA/TMEM63 family channels are osmo- and mechano-sensitive^21,22,25,28,40^. However, the physiological roles of these channels have only begun to be discovered. In Drosophila, TMEM63 channel is responsible for humidity detection and food texture preference^28,29^. In mammals, TMEM63A mutations are associated with hypo-myelination^30^, while TMEM63C is involved in the glomerular filtration barrier function^31^. Our previous work showed that TMEM63B is required for hearing and serves as the osmosensor for cochlear outer hair cells^21^. Our work here further expends our understanding on the physiological roles of TMEM63 family channels and demonstrates that TMEM63B is the long-sought osmosensory molecule in the SFO required for thirst drive^1,2^.

## Materials and methods

### Animals

All animal care and experimental procedures were carried out in accordance with the guidelines for the care and use of laboratory animals and were approved by the Institutional Animal Care and Use Committee at the animal facility of Model Animal Research Center of Nanjing University, Nanjing, China (Protocol no. SOP-LAB-017). Six- to 12-week-old mice of both genders were used in this study. Mice were bred and housed under a condition of the specific-pathogen-free (SPF) on a 12 h light-dark cycle with ad libitum access to food and water unless otherwise noted. Allocation of animal groups was randomly chosen for experiments and all experimental procedures were performed in a blinded manner. *Tmem63b*^*Cre/+*^*;Rosa26*^*mTmG*^ mice were generated by crossing *Tmem63b*^*Cre/+*^ mice with *Rosa26*^*mTmG*^ mice^21^. *Tmem63b* conditional knockout (*Tmem63b*^*HA-fl/HA-fl*^*;Nestin*^*Cre/+*^) (cKO) mice were generated by mating *Tmem63B*^*HA-fl/HA-fl*^ with *Nestin*^*Cre/+*^ mice^22^. *Tmem63b*^*HA-fl/HA-fl*^*;Gad1-GFP* mice were generated by mating *Tmem63B*^*HA-fl/HA-fl*^ mice with *Gad1-GFP* mice^38^. Since the homozygote *Tmem63b*^*−/−*^ mice^21^ and *Tmem63b*^*Cre/Cre*^ mice are postnatally lethal in C57BL/6N background, the mice were bred under FVB/N background for improved survival of the offspring^21^.

### Generation of the Tmem63b^Cre^ mouse line

*Tmem63b*^*Cre*^ knock-in mice were generated by using the CRISPR-Cas9 genome-editing technology. The 5′ arm and 3′ arm of *Tmem63b* gene were amplified from a C57BL/6N mice genome DNA and cloned into polylinkers of a donor plasmid containing *iCre-SV40 PolyA*, and were used as the repair template for homologous recombination. Then, the sgRNAs, linearized donor plasmid and Cas9 protein were co-injected into one-cell fertilized eggs for genetic editing. SgRNA (CCATGCTGCCGTTCTTGC) directs Cas9 endonuclease cleavage in introns 1–2 and create a double-strand break (DSB). Such breaks were repaired by homologous recombination with donor plasmid and *iCre-SV40 PolyA* cassettes were integrated at the first ATG codon of *Tmem63b*. The founder mice were identified by genotyping and followed by sequence analysis. The insertion of iCre-SV40 PolyA into first ATG codon of *Tmem63b* would prevent the transcription of TMEM63B. To examine *Tmem63b* expression level, the total RNAs extracted from mouse brain were reverse-transcribed into cDNAs using HiScript® 1st Strand cDNA Synthesis Kit (Vazyme Biotech, R111) and oligo(dT)18 primer and then a pair of primers were designed to detect *Tmem63b* mRNA in brains from *Tmem63b*^*Cre/+*^ and *Tmem63b*^*Cre/Cre*^ mice; the primer sequences are: 5ʹ-CAGCAGCAACCCGAAGGACTACT-3ʹ (forward) and 5ʹ-TCTCATCGTCCTTTATCCTGAAGAT-3ʹ (reverse).

### Generation of the loxP-lacZ-loxP-HA-Tmem63b mouse line

*LoxP-lacZ-loxP-HA-Tmem63b* knock-in mouse model was generated by inserting *loxP-lacZ-3×SV40 PolyA-loxP* into the site before the coding region of exon 2 of *Tmem63b* gene. Meanwhile, a segment encoding HA was inserted after the signal peptide of *Tmem63b* gene through homologous recombination. The linearized donor vector, the sgRNA (CCTCAACAGCAGCAACCCGA) and Cas9 protein were co-injected into mouse embryos^21^. The founder mice were identified by genotyping and followed by sequence analysis. To verify whether Cre recombinase can remove inserted *lacZ-3×SV40* and rescue the expression of *Tmem63b* gene, a pair of primers with the forward primer base-paired to inserted HA and the reverse one base-paired around exon 6 and 7, was designed to detect *Tmem63b* mRNA in brains by using qRT-PCR; the designed primer sequences are indicated in below: 5ʹ-GATTACGCTGGCTACCCATAC-3ʹ (forward) and 5ʹ-TCTCATCGTCCTTTATCCTGAAGAT-3ʹ (reverse).

### Viral constructs

The following recombinant AAVs were purchased from the BrainVTA:rAAV-CamKIIα-DIO-mCherry-WPRE-hGH-pA (AAV2/5, AAV5 serotype packaged with AAV2 sequence, 5.0 × 10^12^ vg/mL), rAAV-EF1α-DIO-hChR2(H134R)-mCherry-WPRE-hGH-pA (AAV2/5, 3.6 × 10^13 ^ vg/mL), rAAV-CamKIIα-DIO-hM3D(Gq)-mCherry-WPRE-hGH-pA (AAV2/5, 5.3 × 10^12^ vg/mL), rAAV-CamKIIα-DIO-hM4D(Gi)-mCherry-WPRE-hGH-pA (AAV2/5, 5.3 × 10^12^ vg/mL), rAAV-EF1α-DIO-mCherry-WPRE-hGH-pA (AAV2/5, 4.5 × 10^12^ vg/mL), rAAV-CamKIIα-mCherry-WPRE-hGH-pA (AAV2/5, 2.3 × 10^12^ vg/mL), AAV5-hSyn-EGFP-WPRE-hGH-pA (AAV2/5, 5.1 × 10^12^ vg/mL), and AAV-hSyn-EGFP-Cre-WPRE-hGH-pA (AAV2/5, 5.6 × 10^12^ vg/mL). The following recombinant AAVs were purchased from the Vigene Biosciences: AAV-hSyn-DIO-GCaMP6s-P2A-nls-dTomato-hGH-pA (AAV2/5, 2.1 × 10^13^ vg/mL), and rAAV-CamKIIα-Cre-P2A-mCherry-WPRE-hGH-pA (AAV2/5, 2.3 × 10^13^ vg/mL).

### Stereotaxic surgery

Adult mice were anesthetized with a combination of ketamine (1 mg/mL) and xylazine (10 mg/mL), injected intraperitoneally at 10 µL/g bodyweight. Anesthetized mice were fixed on a stereotaxic frame (RWD) equipped with a heating pad and subcutaneously injected with ketoprofen. After an incision made to expose the skull, a small hole with diameter less than 1 mm was made using a hand drill at the region of interest. Viruses were injected with a pulled-glass pipette (tip diameter 20–40 μm) using a micropipette injection system (R480, RWD) at a rate of 100 nL/min. The glass pipette was kept steady for 10 min and then slowly removed. The virus was injected according to the following stereotaxic coordinates: for SFO injections, 250 nL of virus was injected (AP: –0.50 mm; ML: +0 mm; DV: –2.78 mm).

For optogenetic experiments, AAV-EF1α-DIO-hChR2(H134R)-mCherry or AAV-EF1α-DIO-mCherry were injected into the SFO of *Tmem63b*^*Cre/+*^ mice. A 200-µm customized optic fiber (200-μm-diameter core; NA = 0.39; RWD) was placed 300 μm above the virus injection site and attached to the skull with Vetbond and dental cement. For SFO-specific TMEM63B knockout using AAV-Cre virus, mice were given at least 5 weeks of recovery before behavioral tests. The position of stereotaxic injection and fiber implant sites were verified by histology after experiments. Experiment data obtained from individuals with incorrect target sites were excluded from further analysis.

### Behavioral experiments

The licks of fluid were automatically monitored by a customized lickometer (RWD). Mice were acclimated for 10–15 min in the lickometer cage with the spouts providing distilled water and 0.3 M NaCl solutions before the experiments. Two-bottle tests were conducted in the absence of food for 1 h. All behavioral experiments were performed in the light cycle to avoid effects of circadian factors.

For dehydrated thirst experiments, mice were housed in their home cages without water supply but with food for 24 h unless otherwise noted. After water deprivation, mice were acclimatized for 10 min in the lickometer cage without food/water. Then the spouts were automatically opened to test two bottle ingestion behavior.

For osmotic thirst experiments, mice were given an intraperitoneal injection of 2 M NaCl (5 μL/g body weight) or 2 M mannitol solution (10 μL/g body weight). Fluid intake was tested 10 min post hypertonic solute injection without food. For FOS immunostaining, mice were anesthetized and transcardially perfused after water deprivation for 48 h or induction of osmotic thirst for 1 h.

### Optogenetic and chemogenetic experiments

All experiments were performed in lickometer chamber and fluid licks were automatically monitored with a lickometer. Mice were acclimated to the behavioral chamber for 10 min before test. Video was recorded using cameras installed above each cage in some experiments. Experiments were performed during the light cycle.

For brief water access tests, water-satiated mice were given a laser pulses (20-ms, 20-Hz, 10-s) delivered through an optic fiber using a 470 nm laser pulse generator (RWD). The laser intensity was maintained at 10 mW as measured at the tip of the fiber. In each 30-s trial, mice were given ad libitum access to water with manually photostimulated for up to 10 s; each mouse was repeatedly tested for 10 trials with laser pulses on or off. For long time optogenetic test, satiated mice were given ad libitum access to water with photostimulation for 15 min. Photostimulation was delivered for 1-s at 3-s intervals throughout each testing session. For the two-bottle choice test, satiated mice were given ad libitum access to water and 0.3 M NaCl with photostimulation for 15 min.

For chemogenetic activation experiments, followed by an i.p. injection of CNO (1 mg/kg) for 10 min before the behavior test, satiated or dehydrated mice were given access to water or 0.3 M NaCl for 1 h. For chemogenetic inhibition experiments, CNO (10 mg/kg) injection ~20 min prior to behavior test or 2 M NaCl injection, dehydrated or osmotic thirst mice were given access to water or 0.3 M NaCl for 1 h throughout the testing sessions. For immunohistochemistry, after 2 M NaCl injection for 1 h, CNO-treated mice without available water were anesthetized and transcardially perfused.

### Slice calcium imaging

After AAV-hSyn-DIO-GCaMP6s was injected into the SFO of *Tmem63b*^*Cre/+*^ or *Tmem63b*^*Cre/Cre*^ mice for at least 4 weeks, animals were deeply anesthetized with isoflurane in their home cages, and transcardially perfused with an ice-cold oxygenated (95% O_2_/5% CO_2_) cutting solution containing 2.5 mM KCl, 1.25 mM NaH_2_PO_4_, 25 mM NaHCO_3_, 10 mM glucose, 210 mM sucrose, 1.3 mM Na-ascorbic acid, 0.5 mM CaCl_2_, 7 mM MgSO_4_. After decapitation, the brain was immediately removed and submerged in ice-cold cutting solution. Acute coronal slices (200 μm) were sectioned with a vibratome (VT-1000s, Leica) in ice-cold oxygenated cutting solution. Slices were then transferred to oxygenated (95% O_2_/5% CO_2_) artificial cerebrospinal fluid (ACSF) (osmolarity: 300 mOsm/L) containing 119 mM NaCl, 2.5 mM KCl, 26.2 mM NaHCO_3_, 1 mM NaH_2_PO_4_, 11 mM glucose, 2.5 mM CaCl_2_, 1.3 mM MgSO_4_ and incubated at 34 °C for at least 45 min. Slices were then transferred and stored at room temperature at least 1 h until use. The calcium fluorescence imaging was performed using an Olympus epifluorescence microscope. Fluorescent signals were excited with a 470 nm LED and collected through a 20× objective, and digitized with a scientific hamamatsu camera controlled with a HCimage Live software (Hamamatsu Corporation). Calcium fluorescence were imaged at 512 × 512 pixels/frame, 1 frames/5 s, with an exposure time of 800 ms. For image analyses, regions of interest (ROIs) containing the SFO were manually selected in each image stack, and the backgrounds were estimated from the regions where no GCaMP6s was expressed. After background subtraction, the fluorescence ΔF/F was calculated as (stimulated 473 nm signal –F_0_)/F_0_, in which F_0_ is a dynamic baseline fluorescence (F_0_) without hypertonic solution or AngII stimulation. For each neuron, the fluorescent signals >1× standard deviation (SD) of the ΔF/F_0_ of the recording period was selected to study.

### Electrophysiology

Patch-clamp recordings were performed on acute brain slices containing SFO prepared from 6- to 10-week-old mice. After animals were deeply anesthetized and transcardially perfused with an ice-cold oxygenated cutting solution, the brain was immediately removed and acute coronal slices (250 μm) were sectioned with a vibratome (VT-1000s, Leica). The cut slices were then transferred to 34 °C oxygenated (95% O_2_/5% CO_2_) artificial cerebrospinal fluid (ACSF) (osmolarity: 300 mOsm/L) containing 119 mM NaCl, 2.5 mM KCl, 26.2 mM NaHCO_3_, 1 mM NaH_2_PO_4_, 11 mM glucose, 2.5 mM CaCl_2_, 1.3 mM MgSO_4_ and incubated for at least 45 min prior to recording. During experiments, a single slice was placed in a recording chamber to equilibrate for 10 min before recording and continuously perfused with oxygenated ACSF at a rate of 2–3 mL per minute. Neurons of interest were visualized with an upright microscope equipped with infrared-differential interference contrast and fluorescence optics. Synaptic transmission was blocked by 10 μM NBQX, 50 μM D-AP5, 100 μM picrotoxin and 10 μM bicuculline (‘synaptic blockers’). Electrophysiological signals were recorded with whole-cell current-clamp mode at room temperature using an MultiClamp 700B amplifier (Axon Instruments), low-pass filtered at 10 kHz and digitized at 20 kHz using a Digital 1550 (Molecular Devices) and pClamp software (Clampex 10, Axon Instruments). Reported membrane potential values (RMP) were not corrected for the liquid junction potential. Recordings with an access resistance exceeded 30 MΩ were discarded.

For channelrhodopsin validation, optogenetically evoked action potentials and photocurrents were recorded in whole-cell current-clamp configuration and whole-cell voltage-clamp configuration, respectively. Recording pipettes (5–8 MΩ) were pulled from borosilicate glass capillary (O.D. 1.5 mm, I.D. 0.86 mm, RWD) and filled with internal solution (pH: 7.3; osmolarity: 295 mOsm/L) containing 145 mM K-gluconate, 4 mM KCl, 2 mM NaCl, 4 mM Mg_3_ATP_2_, 0.3 mM Na_3_GTP, 0.2 mM EGTA, 10 mM HEPES. Photostimulation (20-Hz; 2-ms pulses) was delivered through a 473-nm laser (RWD) via an optic fiber positioned over the slice. Laser intensity at the end of the optic fiber was measured as 10–12 mW. To test whether CNO (5 μM) effectively caused activation or inhibition of neurons recorded from hM3Dq- or hM4Di-expressing SFO^TMEM63B^ neurons, respectively, CNO was dissolved in ACSF and delivered to slices by local perfusion via a press-driven multichannel system (ALA-scientific) with the outlet placed ~50 μm away from the cell being recorded.

To determine whether SFO neurons showed an increase in firing activity following hyperosmotic solution application, all neurons were patch-clamped in the current-clamp mode and were held at 0 pA. All recordings were done in the presence of synaptic blockers. Hyperosmolarity-induced firing was evoked by perfusion with iso-osmotic (300 mOsm/L) ACSF for 1 min, followed by 2 min of hyperosmotic ACSF solution (+50 mOsm/L mannitol). For hypotonic challenge experiments, the hypoosmotic oxygenated (95% O_2_/5% CO_2_) ACSF (osmolarity: 270 mOsm/L) consisted of: 109 mM NaCl, 2.5 mM KCl, 26.2 mM NaHCO_3_, 1 mM NaH_2_PO_4_, 11 mM glucose, 2.5 mM CaCl_2_, 1.3 mM MgSO_4_. Here, the iso-osmotic 300 mOsm/L ACSF was made by adding 30 mM mannitol into hypoosmotic ACSF without changing ion concentrations. RMP was determined in the presence of 1 μM TTX after a successful patch breakthrough. Average values of firing rate were assessed from the final one minute of the baseline and hypertonic (or hypotonic) stimulation periods, respectively. SFO neurons were classified as stimulation-response if the peak 1 min average firing after stimulation application was greater or lower than the 1 min baseline firing plus 1 SD of the baseline firing rate. To determine the threshold of action potential, SFO^TMEM63B^ neurons were subjected to depolarizing ramp currents. Current ramps were made in ramp (100-ms) from –10 to +20 pA. Threshold was determined at the point where the voltage trace was 10 mV/ms. SFO^TMEM63B^ neuronal input resistance was determined using a series of 500-ms 10-pA increments from –50 pA to 0 pA, calculated according to the slop of current–voltage (I–V) plot for each neuron. An overshoot of at least +20 mV was considered suitable for neurons that were able to fire an action potential. One to two SFO slices were obtained per mouse, and less than 5 SFO neurons were recorded per slice. To determine the current changes induced by hypertonic stimulation in SFO^TMEM63B^ neurons, steady state I–V relationships were analyzed in whole cell-voltage clamp by holding neurons at –70 mV and voltage ramps (800 ms) from –110 to +10 mV were applied every 1 s. Recording pipettes (5–8 MΩ) were filled with internal solution (pH: 7.3; osmolarity: 295 mOsm/L) containing 145 mM CsCl, 2 mM NaCl, 4 mM Mg_3_ATP_2_, 0.3 mM Na_3_GTP, 0.2 mM EGTA, 5 mM QX314-Br, 10 mM HEPES. The changes in slope membrane conductance (G) before and after hyperosmotic solution application were measured from the slope of the I-V relation between –80 and –60 mV. Where indicated, La^3+^ (Sigma) was added at a final concentration of 100 µM. All of the recording currents were filtered at 2 kHz and sampled at 5 kHz.

### Immunohistochemistry and western blotting assays

Mice were anaesthetized with CO_2_ and transcardially perfused with PBS followed by 4% PFA in PBS (pH 7.4). The brain was extracted and post-fixed overnight in PFA at 4 °C. Fixed brain was sectioned into coronal sections at 85-μm thickness using a vibratome (Leica, VT-1000s). After brain sections blocked in a blocking buffer containing 10% donkey serum and 0.2% Triton-X in PBS at room temperature for 1 h, sections were incubated overnight with primary antibodies (rabbit anti-HA, Cell Signaling Technology, 3724, 1:500; rabbit anti-nNOS, Abcam, ab76067, 1:600; goat anti-nNOS, Abcam, ab1376, 1:300; rabbit anti-CamKII, Abcam, ab5683, 1:300; rabbit anti-GAD65/GAD67, ab183999, 1:1000; chicken anti-GFP, Abcam, ab13970, 1:1000; rabbit anti-cFOS, Cell Signaling Technology, 2250, 1:500; chicken anti-Galactosidase, Abcam, ab9361, 1:1000) diluted in blocking buffer overnight at 4 °C. After washed three times (10 min each) with PBS, the sections were incubated in secondary antibodies (Alexa Fluor 594 Donkey anti-chicken, Jackson Immunoresearch, 703-585-155, 1:500; Alexa Fluor 594 Donkey anti-rabbit, Jackson Immunoresearch, 711-585-152, 1:500; Alexa Fluor 647 Donkey anti-rabbit, Jackson Immunoresearch, 711-605-152, 1:500; Alexa Fluor 488 Donkey anti-goat, Jackson Immunoresearch, 705-545-147, 1:500) in blocking buffer for 4 h at room temperature. After washed one time with PBS for 5 min, the sections were incubated in DAPI (2 μg/mL) for 15–20 min. After another three PBS washes, sections were mounted on glass slides and imaged with a Zeiss LSM 880 confocal microscope. Western blotting assay was used to validate the TMEM63B conditional knockout in *Tmem63b*^*HA-fl/HA-fl*^*;Nestin*^*Cre/+*^ mice. Briefly, animals were anaesthetized using isoflurane, and the brain was quickly dissected and homogenized in lysis buffer on ice. After protein concentration was measured by BCA assay, 20 μg protein loaded for each lane was separated on a 10% SDS-PAGE gel and transferred for western blot analysis. The primary antibodies (rabbit anti-HA, Cell Signaling Technology, ab3724, 1:1000; rabbit anti-β-actin, Bioworld, AP0060, 1:10,000), and high-sensitivity ECL reagent (Tanon) were used. The bands were analyzed with Image J.

### Analysis of scRNA-seq data

The scRNA-seq data were downloaded from the Gene Expression Omnibus (GEO) portal (https://www.ncbi.nlm.nih.gov/geo/) (GEO accession no. GSE154048) to analyze the gene expressions of *Tmem63b* and other reported osmosensory and mechanosensitive ion channels and receptors in major cell classes in the SFO. Transcriptomic analysis on cell classes and neuron types was a reference to the previously described analysis method^32^. All additional analyses were performed using the Seurat (3.1.5, http://satijalab.org/seurat/) R toolkit [29608179], including unsupervised clustering and all subsequent analyses.

Briefly, genes expressed in fewer than 20 cells were removed from digital expression data. Finally, a total of 7950 SFO cells with 18,712 genes were used for further analysis. For SFO scRNA-seq datasets, the top 850 variable genes were used for principal component analysis (PCA) to reduce dimensionality. The dimensionality of the scaled integrated data matrix was further reduced to two-dimensional space based on the first 30 principal components (PCs) and visualized by t-Distributed Stochastic Neighbor Embedding (tSNE). The cell clusters were identified based on a shared nearest neighbor (SNN) modularity optimization-based clustering algorithm with a resolution of 1.5. In order to recognize the types of these cells, some known markers, such as *Slc17a6* for Excitatory neurons, *Gad1* for Inhibitory neurons, *Ucma* for LT.astrocytes, *Ntsr2* for Astrocytes, *Plvap* and *Cldn5* for LT.endothelial, *Kcnj8* for Endothelial cells, *Myh11* for Pericytes, *Ccdc153* for Ependymal, *Col1a1* for Fibroblasts, *Fcer1g* for Microglia, *Mag* for Oligodendrocyte, and *Olig2* for Oligo.precursors, were used to verify the annotation of cell types.

### Statistical analysis

Data analysis and generation of histograms were performed using Matlab2018 or Prism8.0, and are described in the figure legends. Results are reported as means ± SEM. Statistical significance was determined by different tests appropriate for each dataset. *P* values for pair-wise comparisons were performed using a two-tailed Student’s *t* test. *P* values for comparing the means of three or more groups were performed using a one-way ANOVA with Bonferroni post hoc tests, or under certain conditions, using a repeated measures two-way ANOVA with Holm-Šídák post hoc tests. Normality of the data distribution was assessed by using the D’Agostino and Pearson omnibus normality test. When normality between sample groups was achieved, one-way ANOVA with Bonferroni’s multiple comparisons tests or two-tailed *t*-tests were used; when normality test failed, two-tailed Mann-Whitney tests were performed. Statistical significance was assigned at *P* < 0.05. *P < 0.05, **P < 0.01, ***P < 0.001, ****P < 0.0001. All tests used for comparisons are specified in the text. No statistical methods were used to predetermine sample sizes. The exact sample size is provided for each experimental group or condition in the text. Analyses were performed blinded to treatment assignments in all behavioral experiments. By pre-established criteria, values were excluded based on histological assessment when the viral injection or optic-fiber implant sites were out of the SFO.

### Supplementary information

Supplementary Information

## Data Availability

The data that support the findings of this study are available from the corresponding author upon reasonable request.
