# Supplementary material for: TMEM63B channel is the osmosensor required for thirst drive of interoceptive neurons
Source: Cell Discov. 2024 Jan 3;10:1. doi: 10.1038/s41421-023-00628-x (PMC10764952; doi:10.1038/s41421-023-00628-x)
Supplement: Supplementary file 1 — Supplementary Information [file 41421_2023_628_MOESM1_ESM.pdf]

Supplementary Information

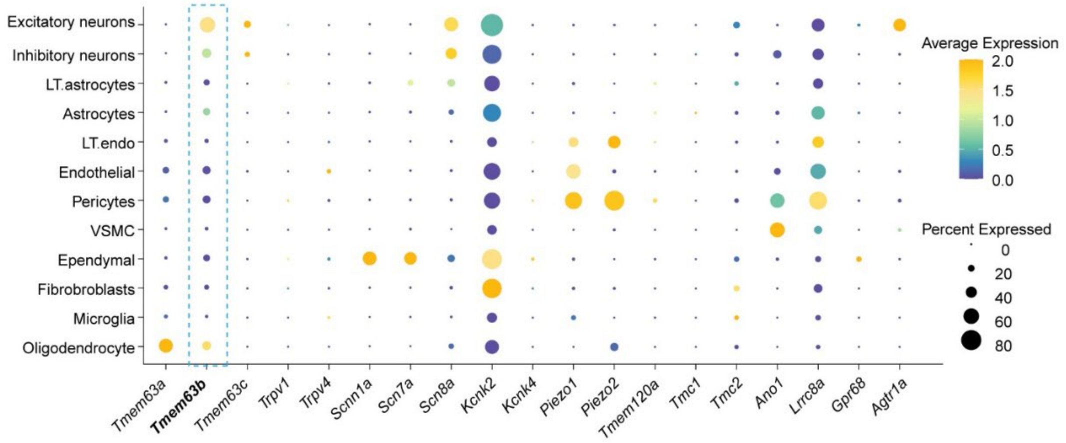

Supplementary Fig. S1 TMEM63B distribution in SFO. Dotplot of cell-type-specific expression for *Tmem63b* and other putative osmosensory and mechanosensitive ion channels and receptors in major cell classes in the SFO. *Agtr1a* (angiotensin II receptor, type 1a) serves as a positive control gene expressed in SFO (dot size is proportional to % of cells with transcript count > 0 expression, colour scale represents z-scored average gene expression, *n* = 7,950 cells for SFO; the scRNA-seq data obtained from the NCBI GEO database, GEO accession no. GSE154048).

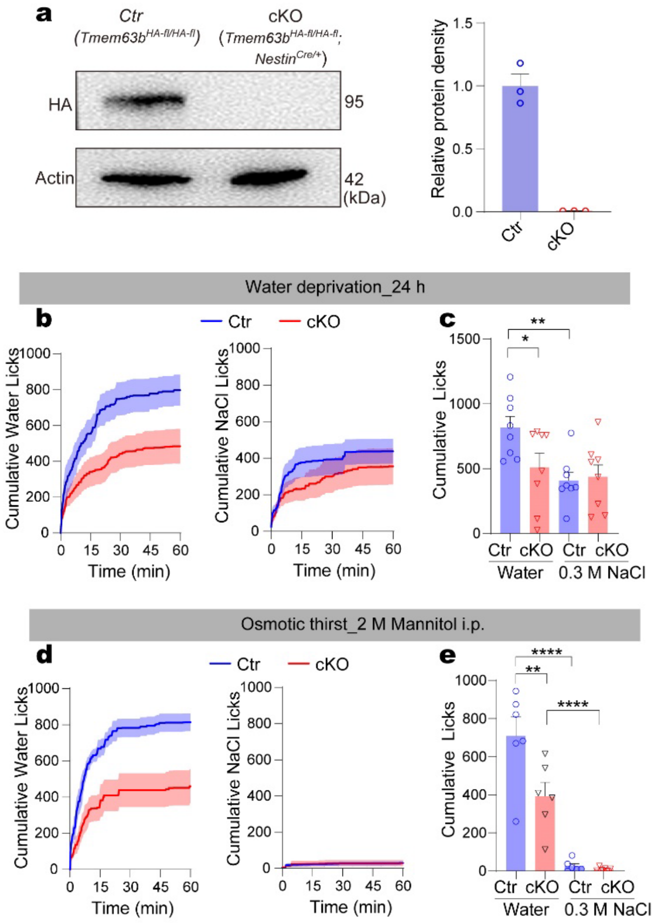

Supplementary Fig. S2 TMEM63B deletion impairs water appetite through a central mechanism. a Western blot validating conditional knockout of central nervous system TMEM63B by using anti-HA antibody. **b, d** Time course of licking after water deprivation (**b**), or mannitol (**d**) injection in *Tmem63B*$^{HA\text{-}fl/HA\text{-}fl}$ (Ctr) and cKO mice. **c, e** Quantification of licking for 60 min after water deprivation (**c**) or mannitol (**e**) injection in Ctr (*n* = 8 mice for water deprivation, and 6 for mannitol) and cKO mice (*n* = 8 mice for water deprivation, and 6 for mannitol). Data are means ± SEM, **P* < 0.05, ***P* < 0.01, *****P* < 0.0001; n.s., no significant; two-way repeated-measures ANOVA with Holm-Šídák post-hoc analysis for (**c**, **e**).

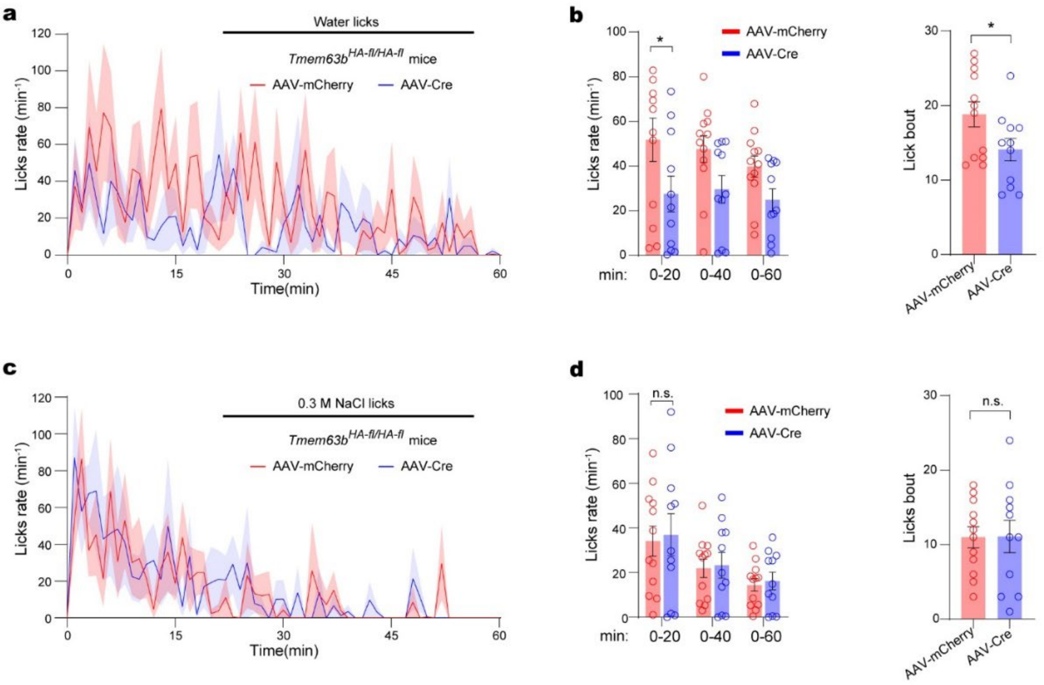

Supplementary Fig. S3 SFO-specific TMEM63B deletion suppressed water intake in water-deprived mice by decreasing lick rate and lick bout. a Time course for the dynamic of water licks (expressed by licks rate) in water-deprived *Tmem63b*$^{HA\text{-}fl/HA\text{-}fl}$ mice injected with AAV-Cre or AAV-mCherry virus. **b** Left: quantification of mean licks rate in different time periods. Right, quantification of lick bout. A lick bout was defined as any set more than 10 licks, in which no inter-lick interval was greater than 5 s. **c, d** Same as (**a**, **b**), but for NaCl licks. Data are means ± SEM, *$P < 0.05$; two-way repeated-measures ANOVA with Holm-Šídák post-hoc analysis and two-tailed unpaired t-test.

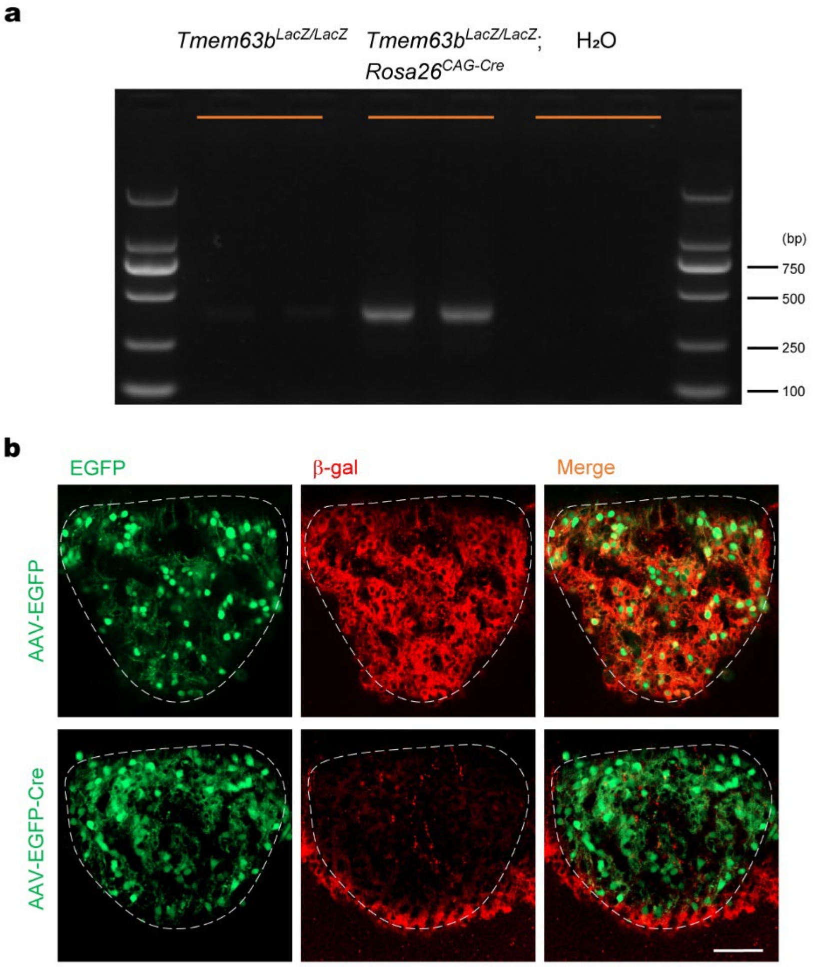

Supplementary Fig. S4 Generation of TMEM63B conditional rescue mice. a RT-PCR was used to validate the expression of *Tmem63b* mRNA in the brain of *Tmem63b*$^{LacZ/LacZ}$ mice and *Tmem63b*$^{LacZ/LacZ}$;*Rosa26*$^{CAG\text{-}Cre}$ mice. The generation of TMEM63B-rescued mice by crossing *Tmem63b*$^{LacZ/LacZ}$ mice with *Rosa26*$^{CAG\text{-}Cre}$ mice; a pair of primers with the forward primer base-paired to inserted HA and the reverse one base-paired around exon 6 and 7, was designed to detect *Tmem63b* mRNA in brains. **b** Representative images validating re-expression of TMEM63B by immunostaining galactosidase (β-gal) after injection of AAV-Cre-EGFP or AAV-EGFP into SFO of *Tmem63b*$^{LacZ/LacZ}$ mice. Scale bar, 50 μm.

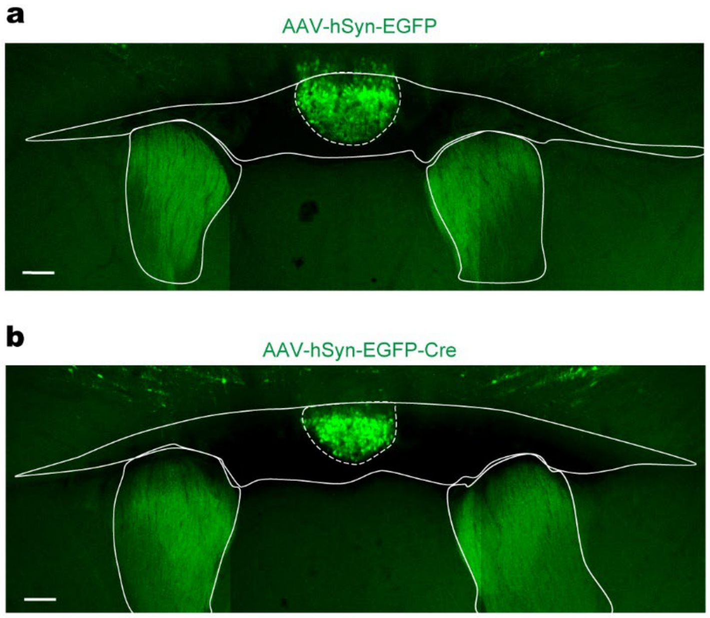

Supplementary Fig. S5 GFP expressing neurons by AAV virus injection was confined in SFO.

a, b Immunochemical detection showing that the virus injection does not travel to other sites in *Tmem63b*$^{LacZ/LacZ}$ mouse, where the GFP expressing neurons were almost restricted in injection site.

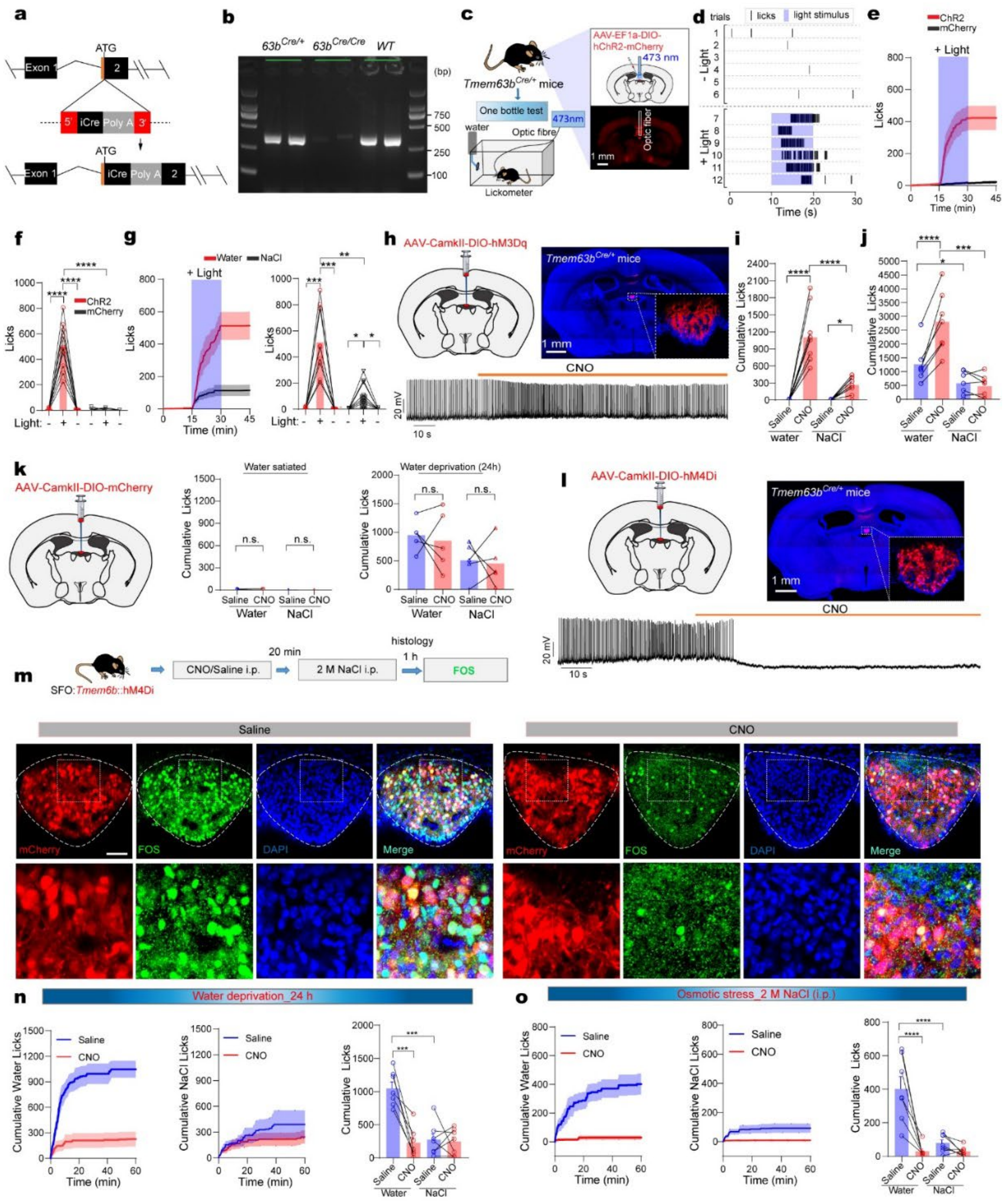

Supplementary Fig. S6 SFO^{TMEM63B} neuron activity encodes thirst. a Schematic illustration showing the strategy used to generate *Tmem63b-iCre* mice. **b** RT-PCR was used to validate the expression of *Tmem63b* in the brain of *Tmem63b$^{cre/+}$* and *Tmem63b$^{cre/Cre}$* mice. **c** Schematic illustration showing the optogenetic activation of SFO^{TMEM63B} neurons. **d** Short time (10 s) photostimulation of SFO^{TMEM63B} neurons (trials 7-12) triggered intense drinking in water-satiated mouse (black lines, licks; blue shading, light stimulus). Each individual trial represented a test last for 30 s and animals were repeatedly tested for 6 trials for each condition, with a minimum intertrial

interval of 60 s. In each 30-s trial (trial 7-12), animals were photostimulated for 10 s to show the efficacy of photostimulation in inducing drinking responses. **e** Long time (15 min) photostimulation efficiently motivates water-satiated mice to drink but not control mice. **f** Quantification (n = 10 for AAV-EF1a-DIO-ChR2 infected mice, and n =7 for AAV-EF1a-DIO-mCherry infected mice). **g** Averaged traces (left) and qualification (right) of water and NaCl licks with optogenetic activation of $SFO^{TMEM63B}$ neurons during the two-bottle test (n = 9 mice). **h** Top: schematic and representative image of AAV-DIO-hM3Dq-mCherry injections. Bottom: representative validation of an hM3Dq-expressing $SFO^{TMEM63B}$ neuron by CNO (5 μM) application. **i, j** Chemogenetic activation of $SFO^{TMEM63B}$ triggered intense water drinking than 0.3 M NaCl drinking under water satiated conditions (**i**) or over drinking under dehydrated conditions but not NaCl over drinking (**j**) (n = 9 mice for water satiated test, 7 for dehydrated thirst test). **k** Control mice infected with AAV-CamKIIa-DIO-mCherry show no more fluid intake after CNO treatment under water satiated or dehydrated conditions (n = 5 mice for each group). **l** Same as (**h**), but mice injected with AAV-DIO-hM4Di-mCherry. **m** Top: experimental design to detect FOS. Bottom: FOS immunoreactivity showing CNO treatment suppresses the activation of SFO neurons triggered by osmotic stress. Scale bar, 50 μm. **n, o** Water deprivation (**n**) or NaCl (i.p.) (**o**)-induced water drinking was significantly suppressed by the chemogenetic inhibition of $SFO^{TMEM63B}$ neurons (n = 7 mice). Data are means ± SEM, *$P < 0.05$, **$P < 0.01$, ***$P < 0.001$, ****$P < 0.0001$, n.s., no significant; two-way repeated-measures ANOVA with Holm-Šídák post-hoc analysis.

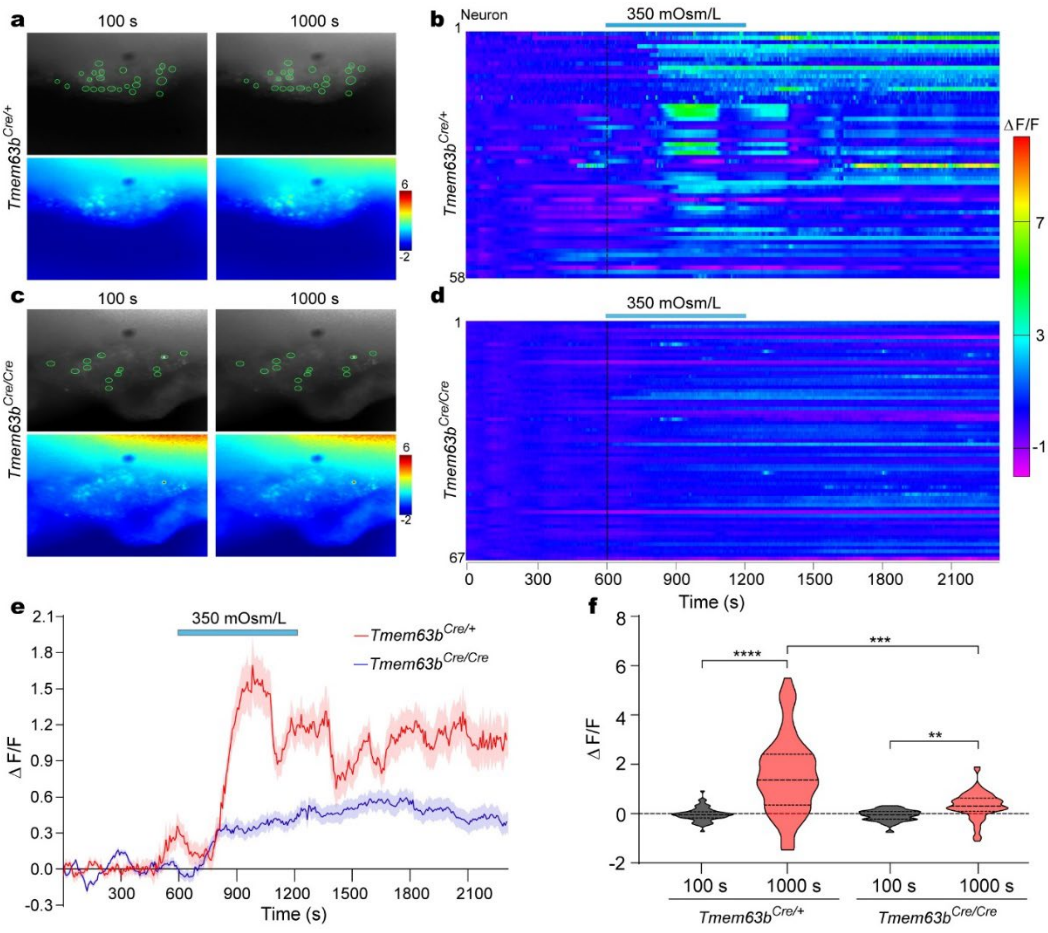

Supplementary Fig. S7 TMEM63B deletion impairs the osmotic sensitivity of thirst neurons in SFO. a, c Representative images from in vitro calcium recordings showing cytosolic Ca^{2+} changes of $SFO^{TMEM63B}$ neurons from AAV-hSyn-DIO-GCaMP6s injected mice before and during application of hypertonic solution. **b, d** Heat map showing the time course of cytosolic Ca^{2+} changes of $SFO^{TMEM63B}$ neurons after application of hypertonic solution (*Tmem63b*$^{Cre/+}$ neurons: $n = 58$ from 2 mice, 2 slices; *Tmem63b*$^{Cre/Cre}$ neurons: $n = 67$ from 2 mice, 2 slices). **e, f** Population Ca^{2+} activity (**e**) and quantification (**f**) from the neurons in (**b**, **d**). Data are means ± SEM, $^{**}P < 0.01$, $^{***}P < 0.001$, $^{****}P < 0.0001$; two-way repeated-measures ANOVA with Holm-Šídák post-hoc analysis for (**f**).

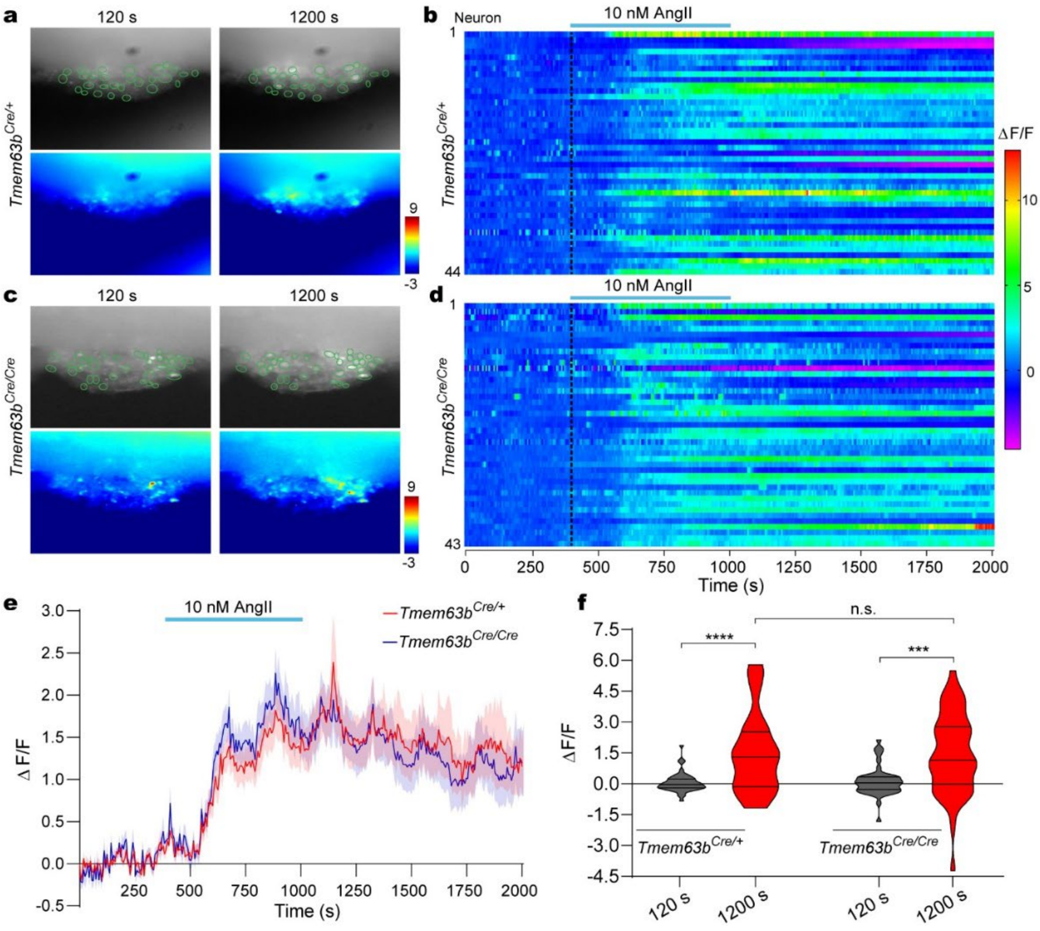

Supplementary Fig. S8 TMEM63B does not affect AngII activation of SFO^{TMEM63B} neurons. a, c Representative images from in vitro calcium recordings showing the effect of AngII on cytosolic Ca^{2+} changes of SFO^{TMEM63B} neurons from AAV-hSyn-DIO-GCaMP6s injected mice. **b, d** Heat map showing the effect of AngII on cytosolic Ca^{2+} changes of SFO^{TMEM63B} neurons (*Tmem63b*$^{Cre/+}$ neurons: n = 44 from 2 mice, 2 slices; *Tmem63b*$^{Cre/Cre}$ neurons: n = 43 from 2 mice, 3 slices). **e** Population Ca^{2+} activity from the neurons in (**b**, **d**). **f** Quantification. Data are means ± SEM, ***P < 0.001, ****P < 0.0001; n.s., no significant; two-way repeated-measures ANOVA with Holm-Šídák post-hoc analysis for (**f**).
